# Comparative mitochondrial genomics of cryptophyte algae: gene shuffling and dynamic mobile genetic elements

**DOI:** 10.1186/s12864-018-4626-9

**Published:** 2018-04-20

**Authors:** Jong Im Kim, Hwan Su Yoon, Gangman Yi, Woongghi Shin, John M. Archibald

**Affiliations:** 1 Department of Biology, Chungnam National University, Daejeon, 34134 South Korea; 2 Department of Biological Sciences, Sungkyunkwan University, Suwon, 16419 South Korea; 3 Department of Multimedia Engineering, Dongguk University, Seoul, 04620 South Korea; 4 Department of Biochemistry and Molecular Biology, Dalhousie University, Halifax, NS B3H 4R2 Canada

**Keywords:** Cryptophytes, Genome re-arrangement, Mitochondrial genome, Mobile genetic elements

## Abstract

**Background:**

Cryptophytes are an ecologically important group of algae comprised of phototrophic, heterotrophic and osmotrophic species. This lineage is of great interest to evolutionary biologists because their plastids are of red algal secondary endosymbiotic origin. Cryptophytes have a clear phylogenetic affinity to heterotrophic eukaryotes and possess four genomes: host-derived nuclear and mitochondrial genomes, and plastid and nucleomorph genomes of endosymbiotic origin.

**Results:**

To gain insight into cryptophyte mitochondrial genome evolution, we sequenced the mitochondrial DNAs of five species and performed a comparative analysis of seven genomes from the following cryptophyte genera: *Chroomonas*, *Cryptomonas*, *Hemiselmis*, *Proteomonas*, *Rhodomonas*, *Storeatula* and *Teleaulax*. The mitochondrial genomes were similar in terms of their general architecture, gene content and presence of a large repeat region. However, gene order was poorly conserved. Characteristic features of cryptophyte mtDNAs included large syntenic clusters resembling α-proteobacterial operons that encode bacteria-like rRNAs, tRNAs, and ribosomal protein genes. The cryptophyte mitochondrial genomes retain almost all genes found in many other eukaryotes including the *nad*, *sdh*, *cox*, *cob*, and *atp* genes, with the exception of *sdh*2 and *atp*3. In addition, gene cluster analysis showed that cryptophytes possess a gene order closely resembling the jakobid flagellates *Jakoba* and *Reclinomonas*. Interestingly, the *cox*1 gene of *R. salina*, *T. amphioxeia*, and *Storeatula* species was found to contain group II introns encoding a reverse transcriptase protein, as did the *cob* gene of *Storeatula* species CCMP1868.

**Conclusions:**

These newly sequenced genomes increase the breadth of data available from algae and will aid in the identification of general trends in mitochondrial genome evolution. While most of the genomes were highly conserved, extensive gene arrangements have shuffled gene order, perhaps due to genome rearrangements associated with hairpin-containing mobile genetic elements, tRNAs with palindromic sequences, and tandem repeat sequences. The *cox*1 and *cob* gene sequences suggest that introns have recently been acquired during cryptophyte evolution. Comparison of phylogenetic trees based on plastid and mitochondrial genome data sets underscore the different evolutionary histories of the host and endosymbiont components of present-day cryptophytes.

**Electronic supplementary material:**

The online version of this article (10.1186/s12864-018-4626-9) contains supplementary material, which is available to authorized users.

## Background

The cryptophyte algae are an evolutionarily significant unicellular eukaryotic lineage inhabiting marine, brackish, and freshwater environments [1, 2]. Cryptophytes are comprised of photosyntheticic, heterotrophic and osmotrophic species [3–9]; phototrophs contain plastids with chlorophyll-*a* and -*c*, as well as phycobilins as accessary pigments. Cryptophytes have a clear phylogenetic affinity to heterotrophic eukaryotes, including goniomonads, kathablepharids and *Palpitomonas bilix*, which collectively have been proposed to comprise a monophyletic assemblage known as Cryptista [10–12]. Cryptophyte cells contain four genomes: host-derived nuclear and mitochondrial genomes, and plastid and nucleomorph genomes of endosymbiotic origin. Given this unusual feature, cryptophytes provide direct evidence for secondary endosymbiosis, a process whereby a photoautotrophic eukaryote is engulfed by a phagotrophic eukaryote [13, 14]. Secondary endosymbiosis has given rise to photosynthetic groups in other protist lineages as well (e.g., euglenoids and chlorarachniophytes from green-algal derived secondary endosymbioses, and cryptophytes haptophytes, stramenopiles, and dinoflagellates, whose plastids are of red-algal origin).

Relatively little is known about mitochondrial diversity in cryptophytes and their non-photosynthetic relatives. Considering eukaryotes as a whole, mitochondrial genomes vary significantly in size, gene content, and genome organization [15–17]. The least derived mitochondrial genomes known are found in an obscure protist group called jakobids and harbor up to 100 genes (including protein-coding genes and genes for noncoding RNA molecules) [18], whereas the mitochondrial genomes of Myzozoa (a subgroup of alveolates) code for only three proteins [19]. In terms of genome architecture, the most common structure is a contiguous circular-mapping DNA molecule, but a contiguous linear genome or genomes segmented into multiple circular or linear molecules are known from some taxa [15, 19]. Although considerable progress has been made in exploring the diversity and evolution of mitochondrial genomes across the full breadth of eukaryote phylogeny, representative genome sequences are still limited or altogether lacking for a large number of eukaryotic lineages, especial protists [16]. Hence, further sampling is necessary to get a full picture of the evolutionary history of mitochondria and their genomes.

To date, only two cryptophyte mitochondrial genomes have been sequenced, those of *Rhodomonas salina* and *Hemiselmis andersenii* [20, 21]. More recently, the mitochondrial genome of *Palpitomonas bilix*, a member of the Cryptista, was sequenced [22]. Here we present the sequences of five mitochondrial genomes belonging to the following organisms: the blue/green-colored cryptophyte *Chroomonas placoidea*, the brown-colored *Cryptomonas curvata* and the red-colored *Proteomonas sulcata*, *Storeatula* sp. CCMP1868 and *Teleaulax amphioxeia*. We performed a comparative analysis of genome structure and gene re-arrangements and investigated the phylogeny of mitochondrial genes relative to nuclear and plastid genes. Our results provide important insights into the broad evolution of organelle genomes and fine-scale dynamics within cryptophytes.

## Results

### General features of cryptophyte mitochondrial genomes

**Fig. 1:**
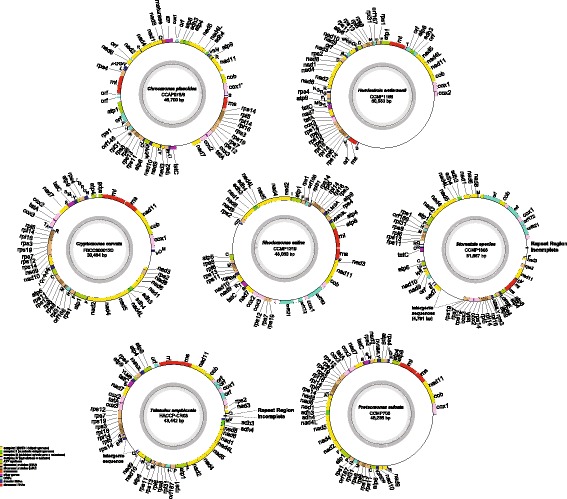
Circular map of the mitochondrial genome of seven cryptophytes. The protein coding genes, rRNA and tRNA genes (single letter) are labeled outside the circle. The genes are color-coded according to the functional categories in the index

Mitochondrial genomes (mtDNAs) were sequenced and analyzed from representatives of three different colored cryptophytes: the red-coloured *Proteomonas*, *Rhodomonas*, *Storeatula* and *Teleaulax*, the green-coloured *Chroomonas* and *Hemiselmis*, and the brown-colored *Cryptomonas* species (Table 1). The mitochondrial genomes of two species, *Storeatula* sp. CCMP1868 and *Teleaulax amphioxeia*, could not be completely assembled, due to the presence of complex repeat regions (Fig. 1), as seen previously (e.g., in the haptophyte *Phaeocystis*; [23]). All of the newly sequenced cryptophyte mitochondrial genomes were found to have a highly repetitive non-coding region (Table 1), as previously described in two other cryptophyte mitochondrial genomes [20, 21]. The mitochondrial genomes ranged in size from ~ 37 Kbp (*Cryptomonas curvata*) to ~ 54.5 Kbp (*Storeatula* sp. CCMP 1868), not including repeat regions (Table 1). They share a core set of 2 rRNAs, 25~ 28 tRNAs and 42 protein-coding genes, including 18 components of the respiratory chain, 5 ATP synthase subunits, 16 ribosomal proteins and 2 subunits of the *tat* translocase (Tables 2, 3). Notably, the mitochondrion-encoded *rps*1 and *tat*A genes in cryptophytes were previously found to be present exclusively in jakobids and/or malawimonads, believed to possess the most ‘primitive’ mitochondrial genomes known [18]. Minor variation was found in tRNA gene content (Table 2). For instance, *trn*K(UUU) is present in three red-colored cryptophytes, *R. salina*, *Storeatula* sp. CCMP1868, and *T. amphioxeia*, but not *Proteomonas sulcata*, while the blue/green-colored *Ch. placoidea* and *H. andersenii* and the brown-colored *Cr. curvata* have a unique isotype *trn*G(GCC) (Table 2).

**Table 1:** Characteristics of cryptophyte mitochondrial genomes analyzed in this study

General characteristics	*Proteomonas sulcata* CCMP705	*Teleaulax amphioxeia* HACCP CR01	*Storeatula* species CCMP1868	*Rhodomonas salina* CCMP1319	*Cryptomonas curvata* FBCC300012D	*Hemiselmis andersenii* CCMP644	*Chroomonas placoidea* CCAP978/8
Plastid color	red	red	red	red	brown	green	green
Genome size (bp)	37,009	43,442	54,527	43,375	37,438	40,898	44,384
(with Repeats)	(48,238)	(unknown)	(unknown))	(48,063)	(39,464)	(60,553)	(46,700)
G + C (%)	31.2	32.8	29.0	29.8	25.81	30.0	29.4
repeat region	not inverted	inverted	inverted	inverted	not inverted	not inverted	not inverted
	present	present	present	present	present	present	present
	(1.1Kb)			(4.7Kb)	(2.03Kb)	(19.7Kb)	(2.3 Kb)
Total gene (include RNAs)	69	73	73	71	71	74	79
Gene direction (+/−)	69/0	42/31	40/33	51/20	29/42	74/0	79/0
No. of protein-coding genes	42	45	43	42	42	44	50
Unknown ORFs	1	4	2	1	1	3	6
tRNAs	25	26	28	27	27	28	27
introns		group II	group II	group II			
Genes with intron (no. of intron within gene)		cox1(2)	cox1(3)/cob(1)	cox1(3)			cox1*(2)
gene fission							cox1 (exon1-IEP-maturase)/cox1 (exon2)
GenBank accession	MG680945	MG680944	MG680943	NC_002572	MG680942	NC_010637	MG680941

**Table 2:** Transfer RNAs (tRNAs) found in cryptophyte mitochondrial genomes

	*Chroomonas placoidea*	*Hemiselmis andersenii*	*Cryptomonas curvata*	*Rhodomonas salina*	*Storeatula* species	*Teleaulax amphioxeia*	*Proteomonas sulcata*
trnA(TGC)	1	1	1	1	1	1	1
trnC(ACA)				1			
trnC(GCA)	1	1	1	1	1	1	1
trnD(GTC)	1	1	1	1	1	1	1
trnE(TTC)	1	1	1	1	1	1	1
trnF(GAA)	1	1	1	1	2	1	1
trnG(GCC)	1	1	1				
trnG(TCC)	1	1	1	1	1	1	1
trnH(GTG)	1	1	1	1	1	1	1
trnI(GAT)	1	2	1	2	1	1	1
trnI(TAT)				1			
trnK(TTT)				1	1	1	
trnfK(TTT)		1					
trnL(GAG)	1	1	1			1	
trnL(TAA)	1	1	1	1	1	1	1
trnL(TAG)	1	1	1	1	1	1	1
trnfM(CAT)		1					
trnM(CAT)	3	1	3	2	3	3	3
trnN(GTT)	1	1	1	1	1	1	1
trnP(TGG)	1	1	1	1	1	1	1
trnQ(TTG)	1	1	1	1	1	1	1
trnR(GCG)	1	1	1	1	1		1
trnR(TCG)	1	1	1	1	1	1	1
trnR(TCT)	1	1	1	1	1	1	1
trnS(GCT)	1	1	1	1	1	1	1
trnS(TGA)	1	1	1	1	1	1	1
trnT(TGT)	1	1	1	1	1	1	1
trnV(TAC)	1	1	1	1	1	1	1
trnW(CCA)	1	1	1	1	1	1	1
trnY(GTA)	1	1	1	1	1	1	1
trnY(ATA)	1	1		1			
Total	28	29	27	28	28	26	25

**Table 3:** Functional protein coding genes in the cryptophyte mitochondrial genome (41 total)

Classification	Genes				
Electron transport and ATP synthesis					
NADH dehydrogenase (complex I) subunits	*nad*1	*nad*2	*nad*3	*nad*4	*nad*4L
	*nad*5	*nad*6	*nad*7	*nad*8	*nad*9
	*nad*10	*nad*11			
Succinate dehydrogenase (complex II) subunits	*sdh*3	*sdh*4 (HAM_72)			
Cytochrome *bc1* complex (complex III) subunits	*cob*				
Cytochrome *c* oxidase (complex IV) subunits	*cox*1	*cox*2	*cox*3		
ATP synthase (complex V) subunits	*atp*1	*atp*4 (ymf39)	*atp*6	*atp*8	*atp*9
Translation					
SSU ribosomal proteins	*rps*1 (orf207)	*rps*2	*rps*3	*rps*4	*rps*7
	*rps*8	*rps*11	*rps*12	*rps*13	*rps*14
	*rps*19				
LSU ribosomal proteins	*rpl*5	*rpl*6	*rpl*14	*rpl*16	*rpl*31 (orf72)
Protein import					
SecY-independent transporters	*tat*A	*tat*C (ymf16)			

**Fig. 2:**
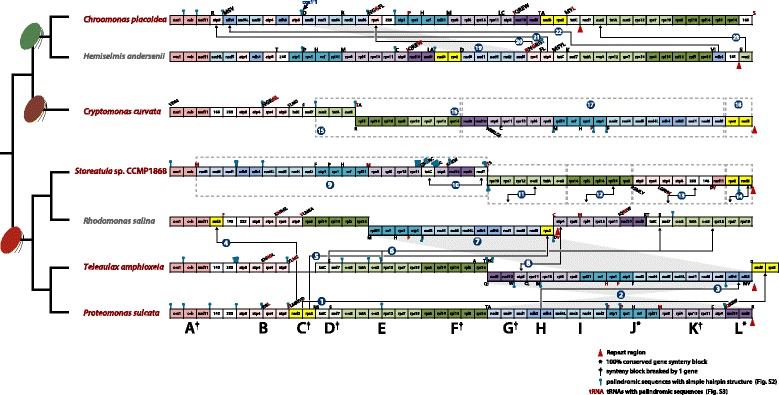
Gene content and arrangement of cryptophyte mitochondrial genomes. Twelve synthetic blocks (A–L) and two rRNAs are described relative to a phylogenetic tree of cryptophytes. Putative gene rearrangements are indicated with numbers (1–23). Tandem repeat regions are marked with red triangles. The most conserved syntenic blocks among cryptophytes are marked; * = 100%, and † = broken by one gene. The hairpin structures with palindromic sequences are marked blue (hairpin symbol). The tRNAs are coded with single letters and tRNAs with palindromic sequences are highlighted red

Further consideration of mitochondrial genome structure revealed that while all genes were located on the same strand in the *Chroomonas placoidea*, *Hemiselmis andersenii* and *Proteomonas sulcata* genomes, some genes were located on the opposite strand in *Cryptomonas curvata* (31 genes), *Storeatula* sp. CCMP1868 (41 genes), *Teleaulax amphioxeia* (39 genes), and *Rhodomonas salina* (53 genes) (Figs. 1, 2, Table 1).

### Genome re-arrangements

The most evident feature of the cryptophyte mitochondrial genomes sequenced herein was that, although gene content is very stable, gene order is highly variable. The 42 protein-coding genes were arranged together and analyzed in the context of 12 syntenic blocks including rRNA and tRNA genes, each block consisting of 2–6 genes (Fig. 2). These blocks were as follows: A) *cox*1*-cob-nad*11, B) *atp*8*-atp*4*-rps*4*-atp*9, C) *nad*3*-rps*2, D) *tat*C*-nad*7, E) *cox*2*-tat*A*-cox*3*-rpl*12*-rpl*7*-rps*19, F) *rps*3*-rpl*16*-rpl*14*-rpl*5*-rps*14, G) *nad*8*-nad*6*-nad*1, H) *sdh*3*-sdh*4, I) *nad*4L*-nad*5*-nad*4*-nad*2, J) *atp*1*-rps*1*-orf*166*, rpl*31, K) *rps*8*-rpl*6*-rps*13*-rps*11*-atp*6, L) *nad10-nad9*, and 2 rRNAs (Fig. 2). Two syntenic blocks were found to be common to all cryptophyte mitochondrial genomes (Fig. 2, J and L).

To assess the extent of mitochondrial genome rearrangements more closely, genomes were aligned using the Mauve genome aligner (Additional file 1: Figure S1). Synteny was broadly conserved within the three differently colored cryptophyte lineages. Overall, we documented 23 instances of mitochondrial genome rearrangements, suggesting that extensive scrambling has occurred since the evolutionary split of these species (Fig. 2 and Additional file 1: Figure S1). Comparing *Proteomonas sulcata* with the three other red-colored cryptophyte mitochondrial genomes, 14 gene-order rearrangements were detected (Fig. 2 ①–⑭). The genes in the mitochondrial genome of *Teleaulax amphioxeia* appear to have been rearranged via transposition of synteny block C (①), an inversion of a set of consecutive genes in blocks G to L (②), and a combination of transposition and inversion involving synteny block H (③). Genes in the mitochondrial genome of *Rhodomonas salina* were rearranged with transposition of *nad*3 and *rps*2 from broken block C (in a manner different than that seen for *T. amphioxeia*) (④, ⑤), transposition of block D–E (⑥), an inversion of a set of consecutive genes in blocks G to J (⑦), and transposition of *atp*6 within block K (⑧). The mitochondrial genome of *Storeatula* sp. CCMP1868 was rearranged with transposition of synteny block G–L (⑨), transposition of *tat*C and *nad*7 from a broken block D (⑩), an inversion of block E (⑪), an inversion of block F (⑫), inversion of block B with rRNAs and *nad*11 being split from block A (⑬), and an inversion of block C (⑭).

In terms of mitochondrial gene order, the brown-colored cryptophyte *Cryptomonas curvata* is more similar to the red-colored lineage than to the blue-greens. Relative to the genome of the red-colored *Proteomonas sulcata*, four gene-order rearrangements were detected (Fig. 2 ⑮–⑱): a combination of an inversion and break in block E–F (⑮–⑯), an inversion of a string of genes in blocks G to L (⑰), and a combination of inversion and transposition block C (⑱). The gene order of blue/green-colored cryptophytes is very different from the other groups. Between the two blue/green-colored cryptophytes *Hemiselmis andersenii* and *Chroomonas placoidea*, five gene-order rearrangements were detected (Fig. 2 ⑲–): transposition of G and partial I block (⑲), transposition of *rps*4 (⑳) and *atp*9 () from broken block B, transposition of *sdh*4 from broken block H (), and transposition of *cox*2 break free from the block E (). Clearly there have been extensive mitochondrial genome rearrangements in each of these lineages during cryptophyte evolution.

### Palindromic sequences and large non-coding regions with tandem repeats

**Fig. 3:**
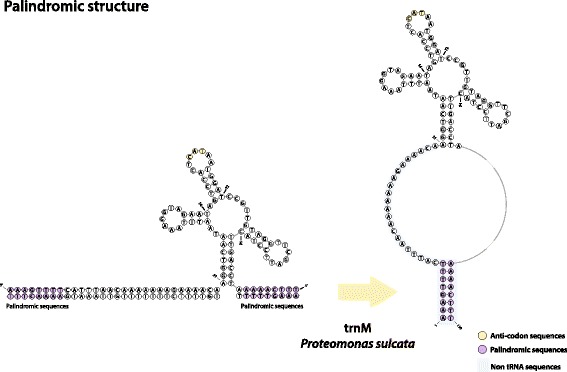
Representative tRNA palindromic sequences in the mitochondrial genome of *Proteomonas sulcata*. The non-tRNA sequences are marked with blue and palindromic sequences are marked with purple. All hairpin structures or tRNAs with palindromic sequences in cryptophtye mitochondrial genome are illustrated in Additional file 2: Figure S2 and Additional file 3: Figure S3

We found between 3 and 20 A-T rich palindromic sequences in the intergenic regions of each of the mitochondrial genomes of cryptophytes (Fig. 3, Additional files 2 and 3: Figures S2 and S3), similar to those reported previously in the large non-coding regions of *Rhodomonas salina* [20] and *Hemiselmis andersenii* [21]. Interestingly, between 4 and 9 tRNA genes are present at the palindromic endpoints (Additional file 3: Figure S3).

Cryptophyte mtDNA structure also appears to have been impacted by the presence of tandem repeats in the large non-coding region, with individual repeats ranging from 1.1Kbp (*P. sulcata*) to 19.7Kbp (*H. andersenii*), each of which contains multi-copy palindromic sequences [20, 21] (Figs. 1 and 2 marked as triangle). The repeats are inverted in the genomes of *R. salina*, *Storeatula* species CCMP1186 and *T. amphioxeia*, but in the other four completely assembled genomes, large non-coding regions are instead dispersed or arranged in tandem throughout the large non-coding region (Table 1).

### Ribosomal proteins and bacterial-like operons

Ribosomal protein gene clusters have been suggested to represent vestiges of bacterial operons in the cryptophyte mitochondrial genome [20]. Our comparisons of mitochondrial gene data provide further support for this idea. The ribosomal protein clusters *rps*3*-rpl*16*-rpl*14*-rpl*5*-rps*14 and *rps*8*- rpl*6*-rps*13*-rps*11 in *Ch. placoidea*, *H. andersenii*, *Cr. curvata*, *R. salina* and *P. sulcata* exhibit the same relative gene order as in the operons of the bacteria *Escherichia coli* and *Rickettsia prowazekii*; this arrangement is also seen (with little variation) in the gene-rich mtDNAs of several other protists (*Jakoba libera, Acanthamoeba catellanii*, *Phytophthora infestans*, and the green alga *Nephroselmis olivacea*) as well as the liverwort *M. polymorpha* (Additional file 4: Figure S4). However, the bacterial-operon cluster *rps*8*- rpl*6*-rps*13*-rps*11 was inverted in the cryptophytes *Storeatula* sp. CCMP 1868 and *T. amphioxeia* (Additional file 4: Figure S4). Furthermore, the other conserved ribosomal protein gene cluster, *rps*12*-rps*7*-rps*19, is translocated in the mitochondrial genome of *Storeatula* sp. CCMP1868.

### Phylogeny

**Fig. 4:**
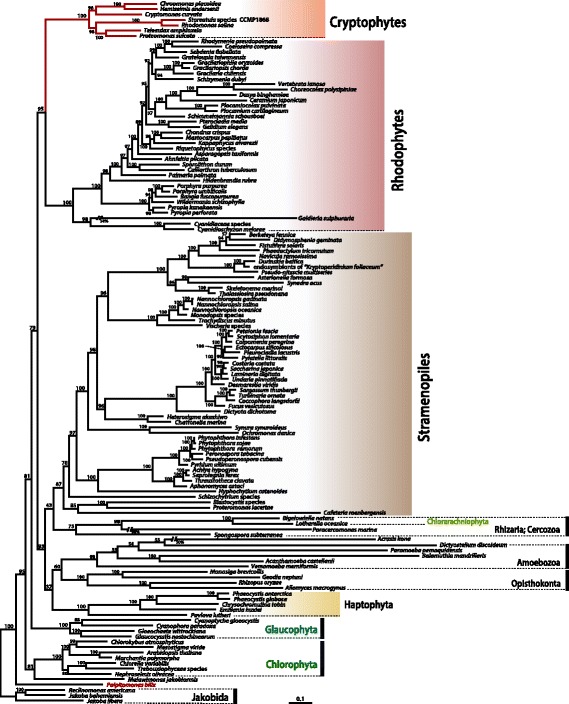
Phylogenetic tree of cryptophyte mitochondrial proteins. The tree was constructed using a dataset of 16 concatenated proteins (4257 amino acids). The numbers on each node represent RAxML bootstrap values. The scale bar indicates the inferred number of substitutions/site

An ML tree was reconstructed using 4257 amino acids from 16 representative genes conserved in the mtDNAs of all chlorophyll-*c* containing algal groups (Fig. 4), as well as diverse photosynthetic and non-photosynthetic lineages from across the eukaryotic tree. The sequences of dinoflagellates and euglenoids were not included in this analysis due to the limited available data of mitochondrial genes. A monophyletic cryptophyte clade was strongly supported (MLB = 100%) and showed well-resolved internal relationships. The four red-colored taxa (i.e., *Storeatula*, *Rhodomonas*, *Teleaulax* and *Guillardia*) group together while the brown-colored *Cryptomonas curvata* forms a sister relationship with the blue/green-colored *Chroomonas* and *Hemiselmis* species (Fig. 4). Although our taxon sampling was limited, these results are consistent with earlier studies. In particular, the red-colored cryptophyte species (*Rhodomonas*, *Storeatula*, *Teleaulax*, and *Proteomonas*) form a monophyletic lineage in both mitochondrial and plastid phylogenomic studies to the exclusion of other cryptophytes ([24], this study), in contrast to single gene analyses of nuclear SSU rDNA [6, 8].

## Discussion

Mitochondrial DNA gene content was found to be essentially identical amongst all cryptophytes examined, although gene order rearrangements were detected. Similar mtDNA architectures, including gene-dense regions, a single large repetitive intergenic region and absolute strand polarity, are seen in the stramenopile *Thraustochytrium aureum* and the green algae *Pedinomonas minor* and *Pycnococcus provasolii* [25, 26]. Structurally speaking, the mitochondrial genomes of haptophycean algae are similar to those of cryptophytes, and show variation of strand polarity within the group. For example, absolute strand polarity is seen in the mtDNAs of *Chrysochromulina tobin* and *Emiliania huxleyi* but not in *Pavlova lutheri* (32/49 genes) and *Phaeocystis globosa* (40/45 genes) [23, 27, 28].

Interestingly, the mitochondrial genomes of various other major algal lineages have also undergone extensive rearrangements. These include the green algae [29, 30], red algae [31], Eustigmatophyceae [32], haptophytes [28], and chlorarachniophytes [33]. The significant structural variation seen in cryptophyte mitochondrial genomes is interesting when compared to the plastid genomes of these same organisms, where gene order was recently shown to be nearly identical [24].

### Palindromic sequences and repeat structures relative to recombination sites

The fact that palindromic sequences are found concentrated near the ends of syntenic blocks in cryptophyte mitochondrial genomes suggests that they have played a role in mediating genomic rearrangements. In fungal mtDNAs, double-hairpin elements have been suggested to act as mobile DNA elements and to mediate lateral gene transfer [34, 35]. In any case, a notable consequence of the presence of such repeats is that they facilitate genome rearrangements [36–39]. A number of possible mechanisms have been proposed. Inversions may occur in a specific location due to the presence of short repeat elements subject to homologous recombination [40, 41]. Even in un-rearranged plastid genomes, small inversions regularly occur in intergenic areas, caused by short inverted repeats forming hairpins that can easily flip the orientation of the intervening sequences [42, 43]. Such a mechanism could explain the highly shuffled genes seen in the cryptophytes mitochondrial genomes described herein (Fig. 2). In addition, tRNA genes associated with gene re-arrangement breakpoints have been reported in plastid genomes [41, 44]. It has been suggested that tRNA genes at inversion endpoints may be due to the presence of short repeats within or near the tRNA genes in the highly rearranged chloroplast genome of the charophyte *Chaetospheridium globosum* [45], as well as in the flowering plant *Trachelium* [46].

The direct repeat arrangements we observed are strikingly similar to the larger (35 kb) repeat structure found in the mitochondrial genome of the diatom *Phaeodactylum tricornutum* [47]. The haptophytes *Chrysochromulina tobin*, *Emiliania hyxleyi*, *Phaeocystis antarctica*, *Phaeocystis globosa* and *Pavlova lutheri*, and the chlorophytes *Pedinomonas minor* and *Acutodesmus obliquus* also contain large tandem repeat regions (>4 kb) in their genomes [23, 25, 27, 28, 48]. In animal mitochondrial genomes, the tandem repeats are located in the control region and help explain the evolutionary origins of tandem repeats among species, populations and even individuals [49, 50]. However, in most algal mitochondrial genomes the tandem repeat manifests itself in a pattern that is species-specific. The repeat sequences within the mitochondrial genomes of cryptophytes described herein do not retain obvious sequence or structural similarity across species bounds.

### Gene content of cryptophyte mitochondrial genomes

**Fig. 5:**
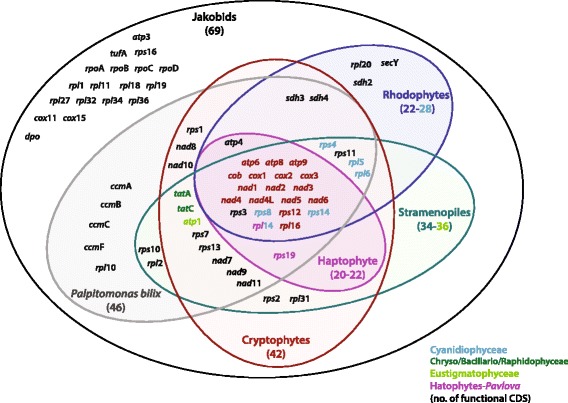
Venn diagram comparing gene content among the mitochondrial genomes of chlorophyll-*c* containg algae. Only genes/proteins with predicted functions are included. Total protein gene numbers are indicated for each group (parentheses). The few genes found in specific subgroups are colored: light-blue in Cyanidiophyceae, green in Chryso/Bacillario/Raphiophyceae, light-green in Eustigmatophyceae, purple in the genus *Pavlova* (Haptophyta). The 16 genes conserved among all groups used to construct phylogenetic trees are highlighted red

The most gene-rich mitochondrial genomes reported to date are those of the excavate group Jakobida, with 61–69 protein-coding genes and 30–34 RNAs [18]. Cryptophyte mitochondrial genomes are also noteworthy in retaining genes that are not found in most other eukaryotes (Fig. 5). The mtDNAs of cryptophytes contain 42 conserved protein genes (excluding non-conserved ORFs in each genome), more than found in glaucophytes (30 CDSs), Bacillariophyceae (34 CDSs), Eustigmatophyceae (36 CDSs), Phaeophyceae (34 CDSs), Rhaphidophyceae (34 CDSs), rhodophytes (22 CDSs), haptophytes (22 CDSs), Synurophyceae (32 CDSs), chlorarachniophytes (26 CDSs) and green algae (11–39 CDSs). Most of the cryptophyte mitochondrial genes are associated with electron transport systems belonging to a set of five complexes, as summarized below.

The genes encoding certain subunits of NADH dehydrogenase (complex I; *nad*7, *nad*8, *nad*9, *nad*10, and *nad*11) are only rarely found in mtDNA. The *nad*7, *nad*9 and *nad*11 genes are missing from all haptophyte and rhodophyte mitochondrial genomes. On the other hand, these three genes are present in all cryptophytes, *Palpitomonas*, stramenopiles and jakobid mitochondrial genomes (Fig. 5). Comparison of the mtDNA gene order in red-/brown-colored cryptophytes reveals the presence of three NADH dehydrogenase gene clusters (cluster G: *nad*8*-nad*6*-nad*1, cluster I: *nad*4L-*nad*5-*nad*4-*nad*2, and cluster L: *nad*10-*nad*9). However, two clusters (G and I) were separated into one or two genes, respectively (G: *nad*8-*nad*1 and *nad*6, cluster I: *nad*4L-*nad*5 and *nad*4-*nad*2), in blue/green-colored cryptophytes (Fig. 2).

The succinate dehydrogenase (complex II) is made up of four protein subunits; two are hydrophilic and belong to the catalytic portion of the complex (*sdh*1 and *sdh*2 genes) and the other two, encoded by the *sdh*3 and *sdh*4 genes, are hydrophobic and act as anchors to the entire complex [51, 52]. The *sdh*2, *sdh*3, and *sdh*4 genes are sometimes found in mitochondrial genomes, whereas *sdh*1 is transferred to the nuclear genome and its protein product is imported from the cytosol. The *sdh*2 gene is present in Rhodophyta except in *Galdieria sulphuraria* [31]. Many other algae have also lost the *sdh*2 gene from their mitochondrial genomes. Mitochondrial genes for two subunits of succinate dehydrogenase (*sdh*3 and *sdh*4) are present in only a few green algae, the liverwort *Marchantia polymorpha*, several red algae [31, 53], all jakobid flagellates including *Reclinomonas americana* [54, 55] and all known cryptophytes ([20, 21], this study).

The genes for subunits of the cytochrome *bc*_*1*_ complex (complex III) and cytochrome *c* oxidase (complex IV) are present in the mtDNAs of all cryptophytes and most algae. The *cob* and *cox*1 genes are always together in all cryptophyte genomes sequenced thus far (Fig. 2A). The *cox*2 and *cox*3 genes are found together in almost all cryptophytes, the exception being the *cox*2 gene in *Hemiselmis andersenii*, which is separated from cluster E (Fig. 2E). Interestingly, the *cox*1 gene of *Storeatula* sp. CCMP1868, *Teleaulax amphioxeia*, and *Rhodomonas salina* was found to contain a putative group II intron with a coding region for an intron encoded protein (IEP). In *Chroomonas placoidea*, the *cox*1 gene was split into two parts with 13 genes in between. The first exon encodes a maturase sequence and is located between the *nad*8-*trn*D and *atp*8 genes. The *cob* gene in *Storeatula* sp. CCMP1868 also has an intron with an IEP (Additional file 5: Figure S5). These introns belong to group II, which often harbor three distinct protein-coding regions corresponding to reverse transcriptase, maturase, and C-terminal DNA binding domains [56]. The *Storeatula* sp. intron coding regions show similarity to an IEP in the *atp*A gene in the mitochondrial genome of the liverwort *Marchantia polymorpha* [57].

The mtDNAs of jakobid flagellates contain the biggest complement of ATP synthase genes (*atp*1, 3, 4, 6, 8, 9). Three *atp* genes (*atp*6, 8, 9) are present in the mitochondrial genomes of green algae, glaucophytes, haptophytes and stramenopiles (Fig. 5). The *atp*1 and *atp*4 genes are present in the mitochondrial genome of green algae, jakobids and cryptophytes. The heterolobosean *Naegleria fowleri* and *Tsukubamonas globose* (a member of the Tsukubamonadidae) have *atp*3 in their mtDNA but lack *atp*4. All cryptophytes and *Palpitomonas bilix* possess most of the *atp* gene set (*atp*1, 4, 6, 8, 9) with the exception of *atp*3 ([22], this study).

The two genes for subunits of the twin-arginine protein translocation system transporters (encoded by the *tat*A and *tat*C genes) are generally conserved in diverse prokaryotes as well as in plastids [58]. The *tat*C homologs are present in some mitochondrial genomes [59], and the mitochondrial *tat*A gene was thought to be limited to the jakobids [60]. However, a recent review of algal mitochondrial genomes [61] noted the presence of *tat*A in some eukaryotic groups, including diatoms, raphidophytes, chrysophytes and cryptophytes; these genes are found in our newly sequenced cryptophyte genome as well (Fig. 5).

### Phylogenetic relationships

Curiously, our 16-protein mitochondrial genome phylogeny shows a strong monophyletic relationship between cryptophytes and rhodophytes. This is unexpected given that the cryptophyte mitochondrion is derived from the host component of the original secondary endosymbiotic partnership that gave rise to modern-day cryptophytes. Based on the monophyletic relationship between chlorophyll-*c* containing groups and red algae seen in many (but not all) plastid genome phylogenies, the hypothesis that a single secondary endosymbiotic uptake of a red alga in a common ancestor of all ‘chromist’ algae has been explored [24, 62–70]. However, recent large-scale studies using nuclear genome data show topologies that are incongruent with this hypothesis. The occurrence of multiple secondary and serial endosymbioses has been proposed to reconcile the apparent incongruence between host and endosymbiont-associated phylogenies (e.g., [12, 69, 71–74]). We examined phylogenies inferred from each of the single genes/proteins used in our multi-gene tree, and could see no consistent signal for a relationship between cryptophytes and rhodophytes to the exclusion of other eukaryotic groups (Additional file 6: Figure S6). In only one of our protein trees (*cob*) did the cryptophytes branch with *Palpitomonas bilix*, to which it is clearly related on the basis of nuclear multi-gene trees (e.g., [10, 12]). Clearly more mitochondrial genome data are needed from plastid-lacking lineages that are related to cryptophytes, such as goniomonads and kathablepharids. Nevertheless, our mitochondrial genome-based phylogeny is consistent with previously published phylogenies, including a 250 nuclear gene dataset [12], in suggesting that the cryptophytes and haptophytes are not specifically related to one other. The monophyly of haptophytes + Opisthokonta/Amoebozoa + Stramenopiles/Rizaria + other relatives is supported by nuclear and mitochondrial analyses ([12], this study).

## Conclusions

We have sequenced mitochondrial genomes from five cryptophyte algae with a wide range of color pigmentation: the red-colored *Proteomonas sulcata*, *Storeatula* sp. CCMP1868 and *Teleaulax amphioxeia*, the blue/green-colored *Chroomonas placoidea*, and the brown *Cryptomonas curvata*. These newly sequenced genomes will aid in the identification of general trends in mitochondrial genome evolution, not just within cryptophytes but between cryptophytes and their closest plastid-lacking relatives such as goniomonads and kathablepharids once these sequence data become available. While most of the cryptophyte genomes we have sequenced are highly conserved with respect to coding capacity, extensive gene arrangements have shuffled gene order. Such rearrangements appear to have been mediated by palindromic sequences, tRNAs, and/or repeat regions. This pattern lies in stark contrast to the high degree of synteny seen in the plastid genomes of these very same organisms, underscoring the dramatic differences in the tempo and mode of organellar genomes of different evolutionary history, even when they reside within the same cell.

## Methods

### DNA isolation and sequencing

Cultures of *Chroomonas placoidea* CCAP 978/8 and *Proteomonas sulcata* CCMP 705 were obtained from the Culture Collection of Algae and Protozoa (CCAP), whereas *Storeatula* species CCMP 1868 came from the National Center for Marine Algae and Microbiota (NCMA). *Teleaulax amphioxeia* collected from Gomso Bay, Korea (35° 40′ N, 126° 40′ E) was established as clonal cultures from single-cell isolates and the strain is available from the Culture collection at the Chungnam National University, Korea. *Cryptomonas curvata* collected from Cheongyang pond, Korea (36° 30′ N, 126° 47′ E) was established as clonal cultures from single-cell isolates and the strain *Cryptomonas curvata* FBCC 300012D is available from the Freshwater Bioresources Culture Collection at the Nakdong-gang National Institute of Biological Resources Korea. All cultures were grown in AF-6 medium [75] with distilled water for the freshwater strain (*Cr. curvata*) or distilled seawater for marine strains, and were maintained at 20 °C under conditions of a 14:10 light:dark cycle with 30 μmol photons·m^− 2^·s^− 1^ from cool white fluorescent tubes. DNA was extracted using the QIAGEN DNEasy Blood Mini Kit (QIAGEN, Valencia, CA, USA) as per manufacturer’s instructions. Next-generation sequencing (NGS) was carried out using the Ion Torrent PGM platform (Thermo Fisher Scientific, San Francisco, California, USA). Sequencing libraries were prepared using the Ion Xpress™ Plus gDNA Fragment Library Preparation Kit for 200 bp or 400 bp-sized sequencing library prearation and the Ion OneTouch™ 200 or 400 Template Kit (Thermo Fisher Scientific, San Francisco, CA, USA) according to the manufacturer’s protocol. Genomes were sequenced on an Ion Torrent Personal Genome Machine (PGM) using the Ion PGM sequencing 200 or 400 Kit (Thermo Fisher Scientific, San Francisco, CA, USA). On the MiSeq (Illumina, San Diego CA), the amplified DNA was fragmented and tagged using the NexteraXT protocol (Illumina), indexed, size selected, and pooled for sequencing using the small amplicon targeted resequencing run, which performs paired end 2 × 300 bp sequencing reads using the MiSeq Reagent Kit v3 (Illumina), according to the manufacturer’s recommendations.

### Genome assembly and annotation

The raw reads obtained from both NGS platforms (i.e., Ion Torrent and Illumina MiSeq) were trimmed separately using the following settings: base = 80 bp, error threshold = 0.05, n ambiguities = 2. Assemblies were also carried out separately. For the Ion Torrent data, the assembly was carried out using MIRA4 (http://mira-assembler.sourceforge.net/docs/DefinitiveGuideToMIRA.html), whereas SPAdes 3.10 (http://bioinf.spbau.ru/spades) was used for Illumina data. For each genome, the two assemblies were compared and the most ‘complete’ assembly was chosen. The mitochondrial origin of the assembled contigs was verified according to the following criteria and Jung et al. [76]: (i) reads were mapped onto the final consensus contig for each cryptophyte mitochondrial genome using Bowtie2 (similarity = 95%, length fraction = 75%; http://bowtie-bio.sourceforge.net/bowtie2/index.shtml) with preset options (sensitive-local: -D 15 -R 2 -N 0 -L 20 -i s,1,0.75); (ii) BLAST searches using commonly known mitochondrial genes against the entire assembly produced hits to these contigs; and (iii) genome sizes consistent with those of other photosynthetic cryptophyte mitochondrial genomes were obtained.

Protein coding genes as well as rRNA and tRNA genes were compiled from all previously sequenced cryptophyte mitochondrial genomes. Preliminary annotation of protein coding genes was performed using GeneMarkS (http://opal.biology.gatech.edu/GeneMark/). The final annotation file was checked in Geneious Pro 10.2.2 (http://www.geneious.com/) using the ORFfinder with genetic code 4 (Protozoan Mitochondrial Code). Predicted ORFs were checked manually and annotated accordingly.

Transfer RNA genes were identified using the tRNAscan-SE version 2.0 server (http://lowelab.ucsc.edu/tRNAscan-SE/) with default settings and the “Mold/Protozoan Mito” model. The rRNA genes were identified by BLASTn searches against a set of known, previously published mitochondrial rRNA sequences of cryptophytes. To determine intron types, mitochondrial genomes were submitted to the RNAweasel server (http://megasun.bch.umontreal.ca/cgi-bin/RNAweasel/RNAweaselInterface.pl). Physical maps were designed with the OrganellarGenomeDRAW program (http://ogdraw.mpimp-golm.mpg.de/).

Palindromic sequence elements were detected using the EMBOSS explorer (http://emboss.bioinformatics.nl/cgi-bin/emboss/palindrome) with the following parameters: 8 for minimum length of palindromes, 100 for maximum length of palindromes, 10 for maximum gap between elements, and no mismatches within the palindrome. To find palindromic sequences in tRNAs, the maximum gap between elements was set at 100 bp (due to the normal tRNA gene length of less 90 bp). Some detected sequences were manually excluded if a loop region was longer than a stem region. Genome sequences were deposited into the NCBI GenBank database under the following accession numbers (Table 1).

### Gene arrangement comparisons

Two previously published cryptophyte mitochondrial genome sequences were downloaded from GenBank [20, 21]. For structural and synteny comparisons, the genomes were aligned using the Mauve Genome Alignment tool version 2.2.0 [77] with default settings. For the purposes of visualization, we arbitrarily designated the beginning of the *cox1* gene as position 1 in each genome (pointing in the direction of *cob*).

### Phylogenetic analysis

Phylogenetic trees were constructed from datasets created by combining amino acid sequences corresponding to 16 protein coding genes from 131 mitochondrial genomes, including those of seven cryptophytes, five haptophytes, 38 stramenopiles, and 36 red algae (Additional file 7: Table S1). The 16 genes are as follows: *atp*6, *atp*8, *atp*9, *cob*, *cox*1, *cox*2, *cox*3, *nad*1, *nad*2, *nad*3, *nad*4, *nad*4L, *nad*5, *nad*6, *rps*12, and *rpl*16. The dataset was concatenated (4257 amino acid positions) and final refinements were performed by eyes in the MacGDE2.5 program [78].

Maximum likelihood (ML) phylogenetic analyses were performed using RAxML version 8.0.0 [79] with the Le and Gascuel gamma (LG + GAMMA) model [80] for amino acid data chosen by ProtTest 3 [81]. We used 1000 independent tree inferences using the -# option to identify the best tree. The model parameters with gamma correction values and the proportion of invariable sites in the combined dataset were obtained automatically by the RAxML program. Bootstrap support values (MLBS) were calculated using 1000 replicates with the same substitution model.

## Additional files

Additional file 1: **Figure S1.** Overview of cryptophyte mitochondrial genomes. Linearized maps of the five novel complete mitochondrial genomes are compared to those from previous studies. The color coded syntenic blocks are shown above each genome, and the gene maps are shown below. The syntenic blocks above the horizontal line are on the same strand, and those below the line are on the opposite strand. The horizontal bars inside the syntenic blocks show sequence conservation. The block boundaries correspond to sites where inversion events have occurred. In the gene maps, the genes above the horizontal line are transcribed from left to right, and those below the horizontal line are transcribed from right to left. The rRNA genes are shown in red. (PDF 1860 kb)

Additional file 2: **Figure S2.** Hairpin structures wih palindromic sequence in cryptophyte mitochondrial genomes. The non-tRNA sequences are marked with blue and palindromic sequences are marked with purple. (PDF 897 kb)

Additional file 3: **Figure S3.** All tRNAs wih palindromic sequences in cryptophyte mitochondrial genomes. The non-tRNA sequences are marked with blue and palindromic sequences are marked with purple. (PDF 1644 kb)

Additional file 4: **Figure S4.** Conservation of ribosomal protein gene organization. Gene order found in cryptophyte mitochondrial genomes compared with that of the contiguous bacterial str, S10 spec and alpha operons of *Escherichia coli* and *Rickettsia prowazekii*. (PDF 548 kb)

Additional file 5: **Figure S5.** Group II introns in cryptophyte *cox1* and *cob* mitochondrial genes. (PDF 352 kb)

Additional file 6: **Figure S6.** Phylogenetic trees inferred from single genes. The trees were constructed using amino acid sequences of 16 genes: *atp*6, *atp*8, *atp*9, *cob*, *cox*1, *cox*2, *cox*3, *nad*1, *nad*2, *nad*3, *nad*4, *nad*4L, *nad*5, *nad*6, *rps*12, and *rpl*16. The numbers on each node represent ultrafast bootstrap approximation (UFBoot) using IQ-Tree. The scale bars indicate the number of substitutions/site. (PDF 2672 kb)

Additional file 7: **Table S1.** The concatenated data set of 16 protein coding genes for phylogenetic reconstruction. (XLSX 22 kb)

## Abbreviations

CDS: Coding sequences
IEP: Intron encoded protein
ML: Maximum likelihood
MLB: Maximum likelihood bootstrap
mtDNAs: mitochondrial genomes
NGS: Next-generation sequencing
ORF: Open reading frame
PGM: Personal Genome Machine
