# Supplementary material for: Comparative mitochondrial genomics of cryptophyte algae: gene shuffling and dynamic mobile genetic elements
Source: BMC Genomics. 2018 Apr 20;19:275. doi: 10.1186/s12864-018-4626-9 (PMC5910586; doi:10.1186/s12864-018-4626-9)
Supplement: Supplementary file 1 — Figure S1. Overview of cryptophyte mitochondrial genomes. Linearized maps of the five novel complete mitochondrial genomes are compared to those from previous studies. The color coded syntenic blocks are shown above each genome, and the gene maps are shown below. The syntenic blocks above the horizontal line are on the same strand, and those below the line are on the opposite strand. The horizontal bars inside the syntenic blocks show sequence conservation. The block boundaries correspond to sites where inversion events have occurred. In the gene maps, the genes above the horizontal line are transcribed from left to right, and those below the horizontal line are transcribed from right to left. The rRNA genes are shown in red. (PDF 1860 kb) [file 12864_2018_4626_MOESM1_ESM.pdf]

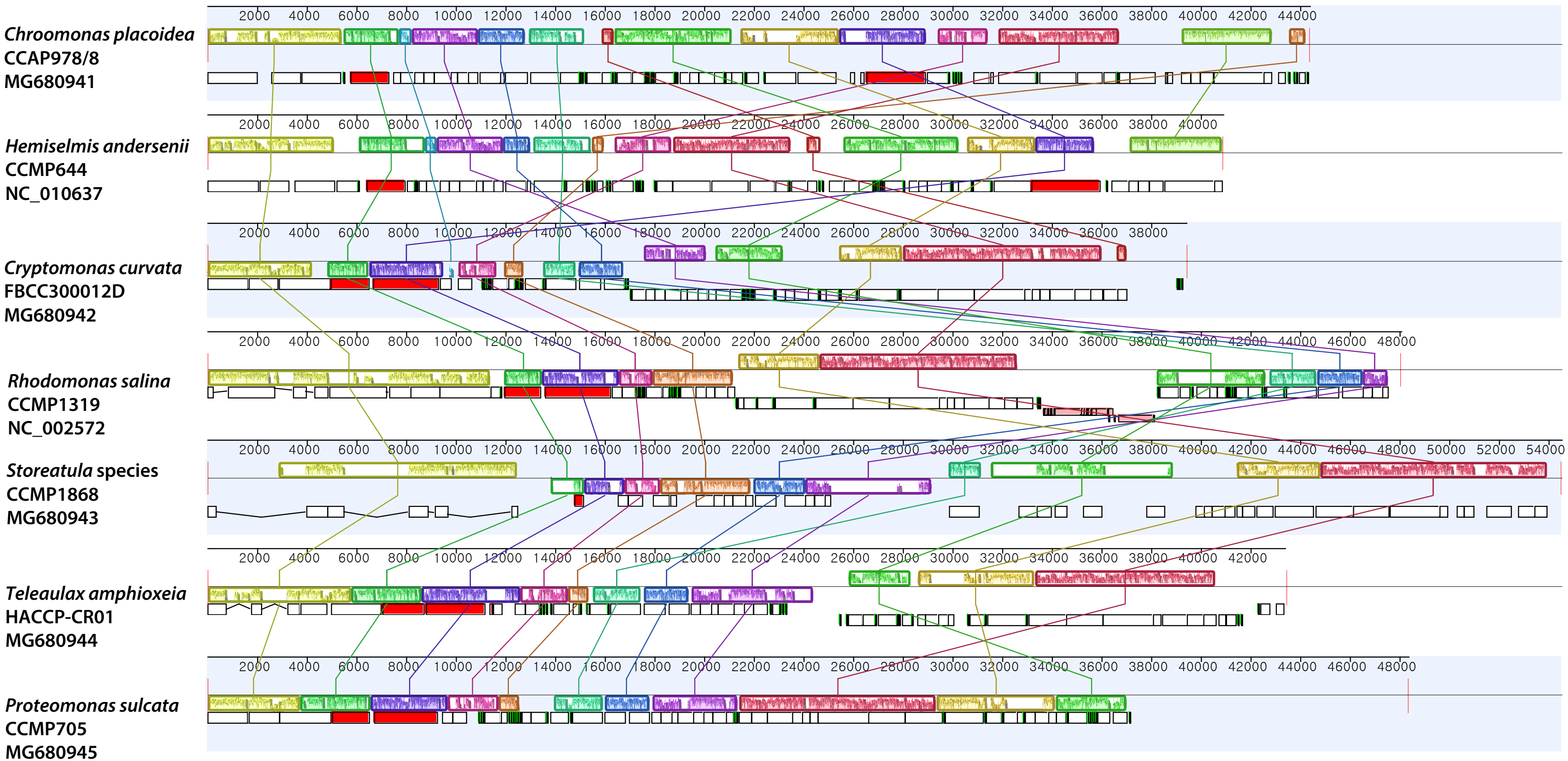
Chroomonas placoidea CCAP978/8 MG680941
Hemiselmis andersenii CCMP644 NC_010637
Cryptomonas curvata FBCC300012D MG680942
Rhodomonas salina CCMP1319 NC_002572
Storeatula species CCMP1868 MG680943
Teleaulax amphioxeia HACCP-CR01 MG680944
Proteomonas sulcata CCMP705 MG680945
