# Supplementary material for: Comparative mitochondrial genomics of cryptophyte algae: gene shuffling and dynamic mobile genetic elements
Source: BMC Genomics. 2018 Apr 20;19:275. doi: 10.1186/s12864-018-4626-9 (PMC5910586; doi:10.1186/s12864-018-4626-9)
Supplement: Supplementary file 2 — Figure S2. Hairpin structures wih palindromic sequence in cryptophyte mitochondrial genomes. The non-tRNA sequences are marked with blue and palindromic sequences are marked with purple. (PDF 897 kb) [file 12864_2018_4626_MOESM2_ESM.pdf]

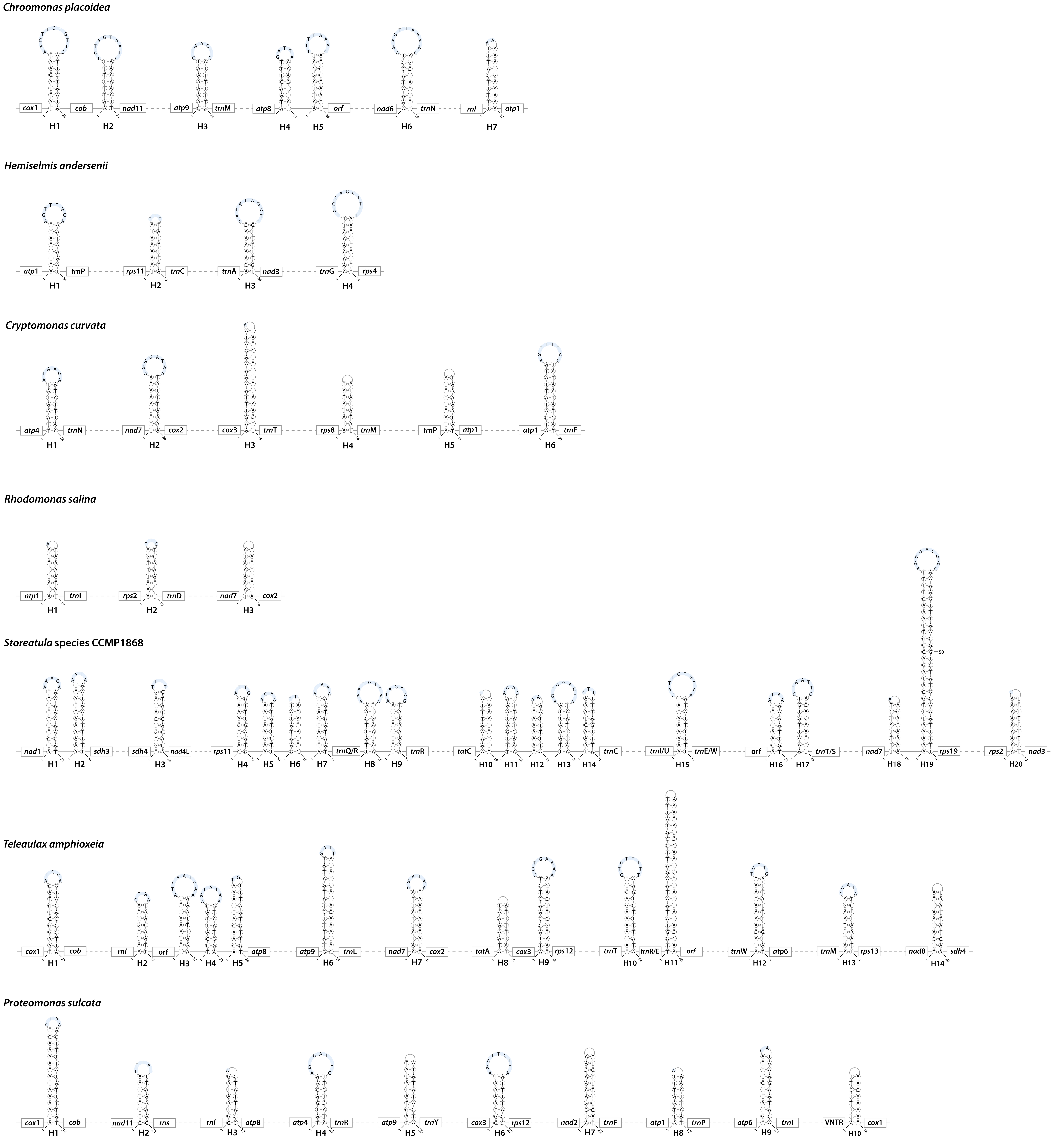
Chroomonas placoidea
cox1 H1 cob H2 nad11 atp9 H3 trnM atp8 H4 H5 orf nad6 H6 trnN rnl H7 atp1
Hemiselmis andersenii
atp1 H1 trnP rps11 H2 trnC trnA H3 nad3 trnG H4 rps4
Cryptomonas curvata
atp4 H1 trnN nad7 H2 cox2 cox3 H3 trnT rps8 H4 trnM trnP H5 atp1 atp1 H6 trnF
Rhodomonas salina
atp1 H1 trnI rps2 H2 trnD nad7 H3 cox2
Storeatula species CCMP1868
nad1 H1 H2 sdh3 sdh4 H3 nad4L rps11 H4 H5 H6 H7 trnQ/R H8 H9 trnR tatC H10 H11 H12 H13 H14 trnC trnI/U H15 trnE/W orf H16 H17 trnT/S nad7 H18 H19 rps19 rps2 H20 nad3
Teleaulax amphioxeia
cox1 H1 cob rnl H2 orf H3 H4 H5 atp8 atp9 H6 trnL nad7 H7 cox2 tatA H8 cox3 H9 rps12 trnT H10 trnR/E H11 orf trnW H12 atp6 trnM H13 rps13 nad8 H14 sdh4
Proteomonas sulcata
cox1 H1 cob nad11 H2 rns rnl H3 atp8 atp4 H4 trnR atp9 H5 trnY cox3 H6 rps12 nad2 H7 trnF atp1 H8 trnP atp6 H9 trnI VNTR H10 cox1
