# Supplementary material for: Comparative mitochondrial genomics of cryptophyte algae: gene shuffling and dynamic mobile genetic elements
Source: BMC Genomics. 2018 Apr 20;19:275. doi: 10.1186/s12864-018-4626-9 (PMC5910586; doi:10.1186/s12864-018-4626-9)
Supplement: Supplementary file 3 — Figure S3. All tRNAs wih palindromic sequences in cryptophyte mitochondrial genomes. The non-tRNA sequences are marked with blue and palindromic sequences are marked with purple. (PDF 1644 kb) [file 12864_2018_4626_MOESM3_ESM.pdf]

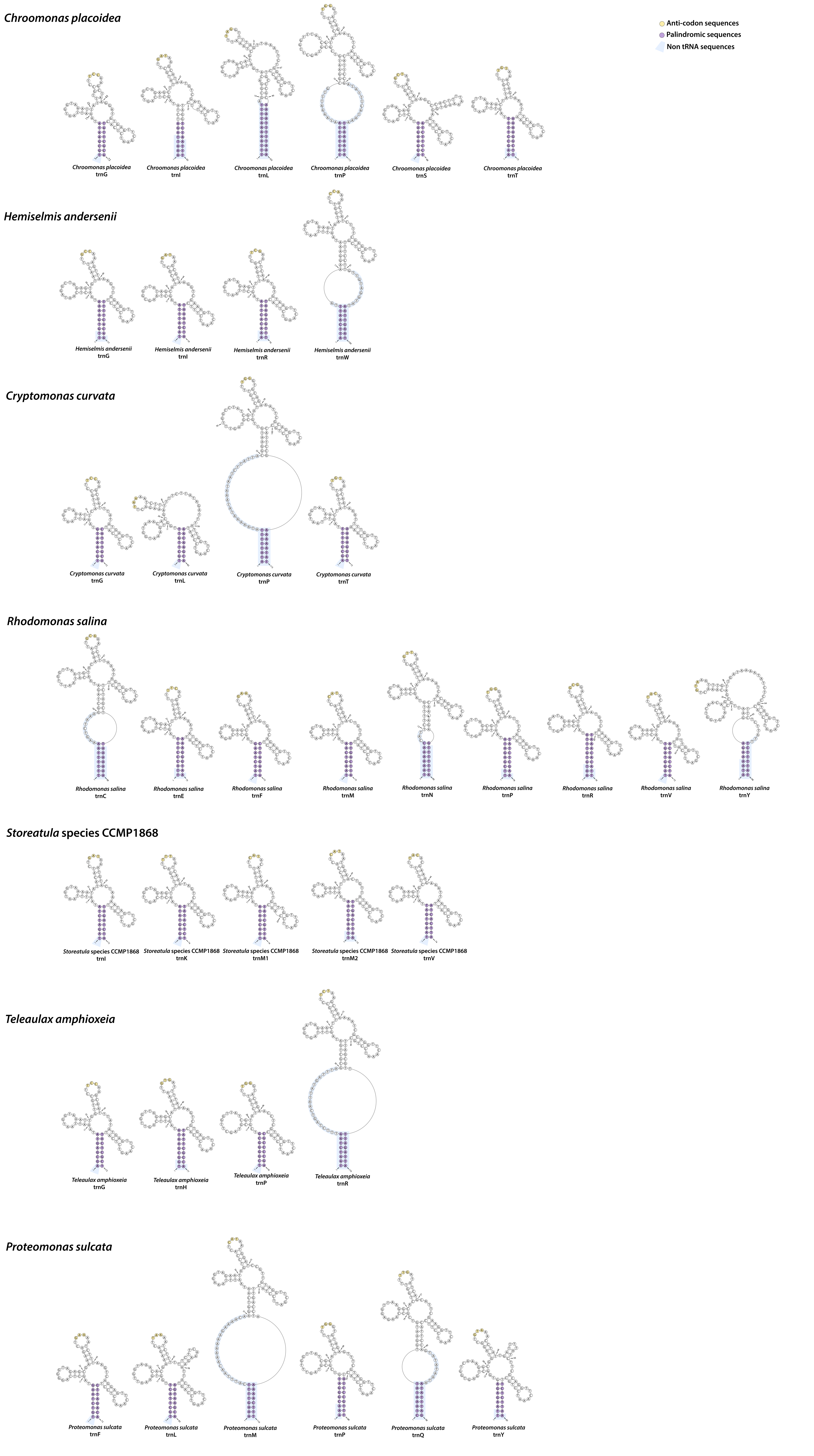
Anti-codon sequences
Palindromic sequences
Non tRNA sequences
Chroomonas placoidea
Chroomonas placoidea trnG
Chroomonas placoidea trnI
Chroomonas placoidea trnL
Chroomonas placoidea trnP
Chroomonas placoidea trnS
Chroomonas placoidea trnT
Hemiselmis andersenii
Hemiselmis andersenii trnG
Hemiselmis andersenii trnI
Hemiselmis andersenii trnR
Hemiselmis andersenii trnW
Cryptomonas curvata
Cryptomonas curvata trnG
Cryptomonas curvata trnL
Cryptomonas curvata trnP
Cryptomonas curvata trnT
Rhodomonas salina
Rhodomonas salina trnC
Rhodomonas salina trnE
Rhodomonas salina trnF
Rhodomonas salina trnM
Rhodomonas salina trnN
Rhodomonas salina trnP
Rhodomonas salina trnR
Rhodomonas salina trnV
Rhodomonas salina trnY
Storeatula species CCMP1868
Storeatula species CCMP1868 trnI
Storeatula species CCMP1868 trnK
Storeatula species CCMP1868 trnM1
Storeatula species CCMP1868 trnM2
Storeatula species CCMP1868 trnV
Teleaulax amphioxeia
Teleaulax amphioxeia trnG
Teleaulax amphioxeia trnH
Teleaulax amphioxeia trnP
Teleaulax amphioxeia trnR
Proteomonas sulcata
Proteomonas sulcata trnF
Proteomonas sulcata trnL
Proteomonas sulcata trnM
Proteomonas sulcata trnP
Proteomonas sulcata trnQ
Proteomonas sulcata trnY
