# Supplementary material for: Comparative mitochondrial genomics of cryptophyte algae: gene shuffling and dynamic mobile genetic elements
Source: BMC Genomics. 2018 Apr 20;19:275. doi: 10.1186/s12864-018-4626-9 (PMC5910586; doi:10.1186/s12864-018-4626-9)
Supplement: Supplementary file 4 — Figure S4. Conservation of ribosomal protein gene organization. Gene order found in cryptophyte mitochondrial genomes compared with that of the contiguous bacterial str, S10 spec and alpha operons of Escherichia coli and Rickettsia prowazekii. (PDF 548 kb) [file 12864_2018_4626_MOESM4_ESM.pdf]

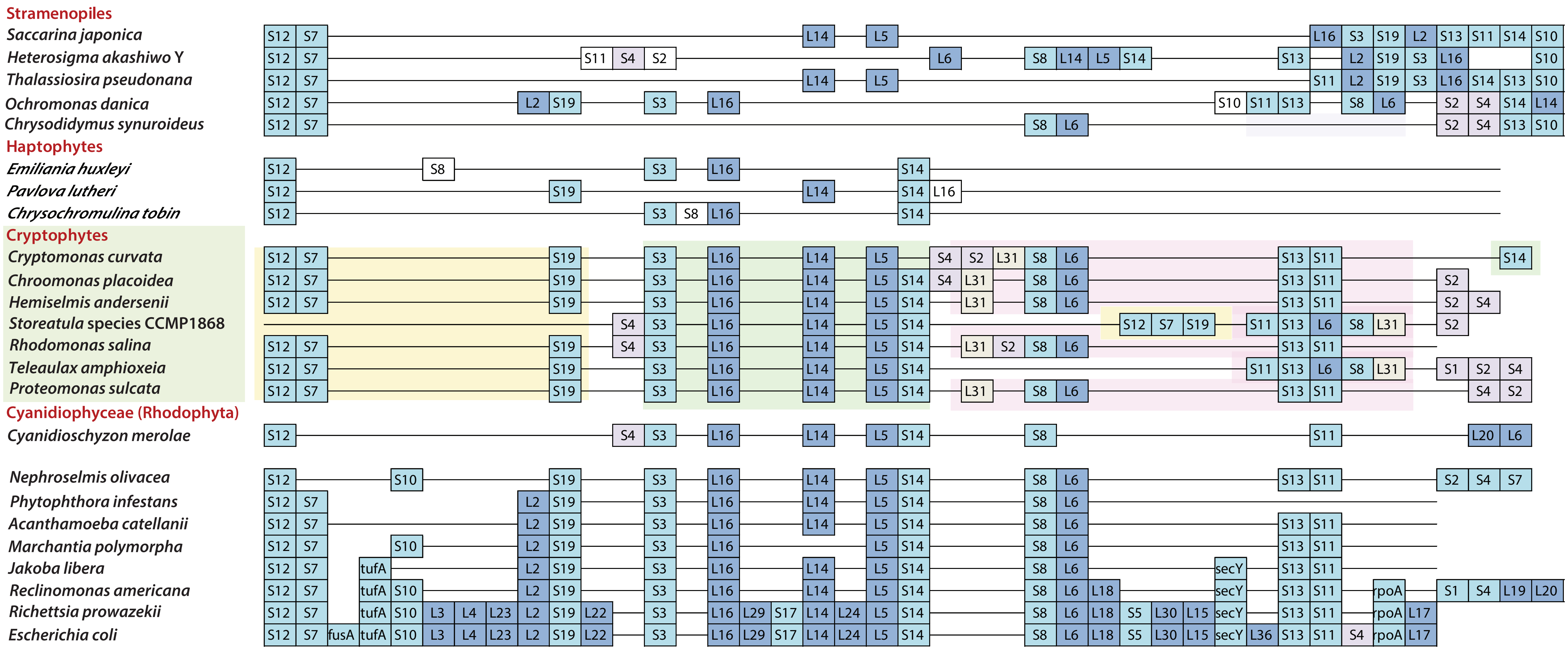

Stramenopiles
Saccarina japonica
Heterosigma akashiwo Y
Thalassiosira pseudonana
Ochromonas danica
Chrysodidymus synuroideus
Haptophytes
Emiliania huxleyi
Pavlova lutheri
Chrysochromulina tobin
Cryptophytes
Cryptomonas curvata
Chroomonas placoidea
Hemiselmis andersenii
Storeatula species CCMP1868
Rhodomonas salina
Teleaulax amphioxeia
Proteomonas sulcata
Cyanidiophyceae (Rhodophyta)
Cyanidioschyzon merolae
Nephroselmis olivacea
Phytophthora infestans
Acanthamoeba catellanii
Marchantia polymorpha
Jakoba libera
Reclinomonas americana
Richettsia prowazekii
Escherichia coli
