# Supplementary figures and images for: Comparative mitochondrial genomics of cryptophyte algae: gene shuffling and dynamic mobile genetic elements
Source: BMC Genomics. 2018 Apr 20;19:275. doi: 10.1186/s12864-018-4626-9 (PMC5910586; doi:10.1186/s12864-018-4626-9)

Cox I

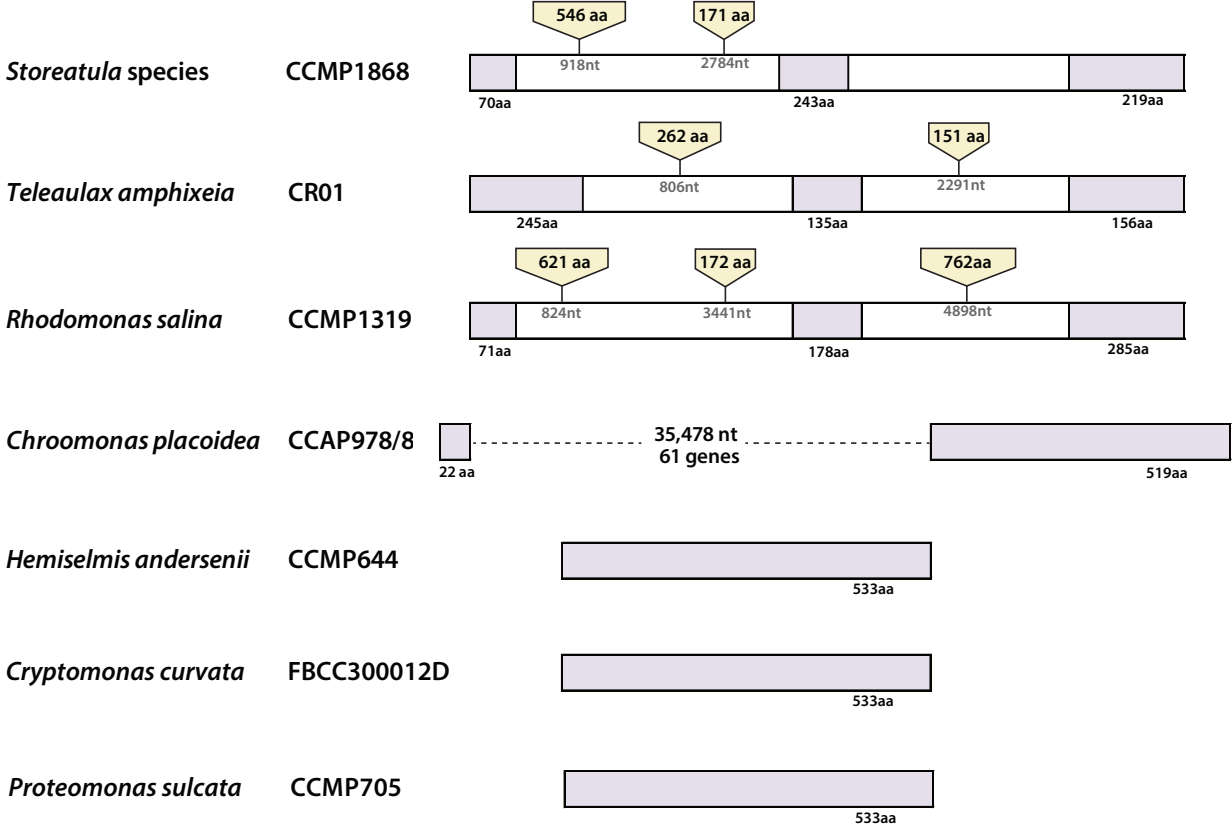

Cob

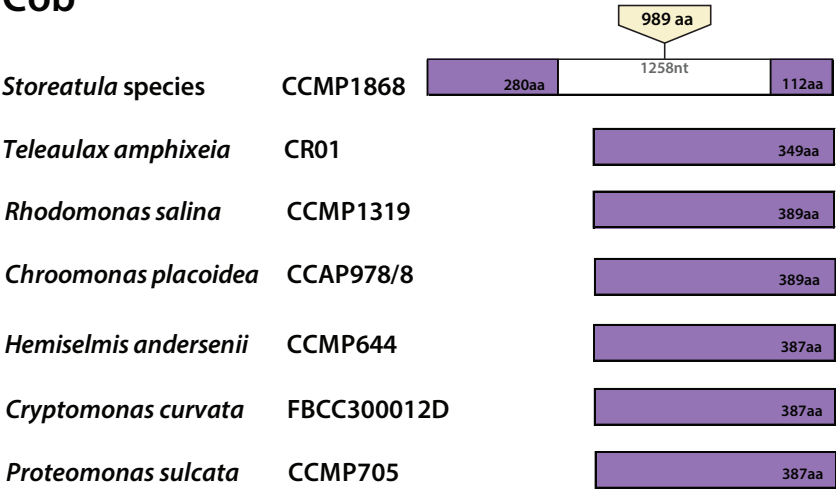

Supplement: Supplementary file 5 — Figure S5. Group II introns in cryptophyte cox1 and cob mitochondrial genes. (PDF 352 kb) [file 12864_2018_4626_MOESM5_ESM.pdf]
