# Supplementary material for: Comparative mitochondrial genomics of cryptophyte algae: gene shuffling and dynamic mobile genetic elements
Source: BMC Genomics. 2018 Apr 20;19:275. doi: 10.1186/s12864-018-4626-9 (PMC5910586; doi:10.1186/s12864-018-4626-9)
Supplement: Supplementary file 6 — Figure S6. Phylogenetic trees inferred from single genes. The trees were constructed using amino acid sequences of 16 genes: atp6, atp8, atp9, cob, cox1, cox2, cox3, nad1, nad2, nad3, nad4, nad4L, nad5, nad6, rps12, and rpl16. The numbers on each node represent ultrafast bootstrap approximation (UFBoot) using IQ-Tree. The scale bars indicate the number of substitutions/site. (PDF 2672 kb) [file 12864_2018_4626_MOESM6_ESM.pdf]

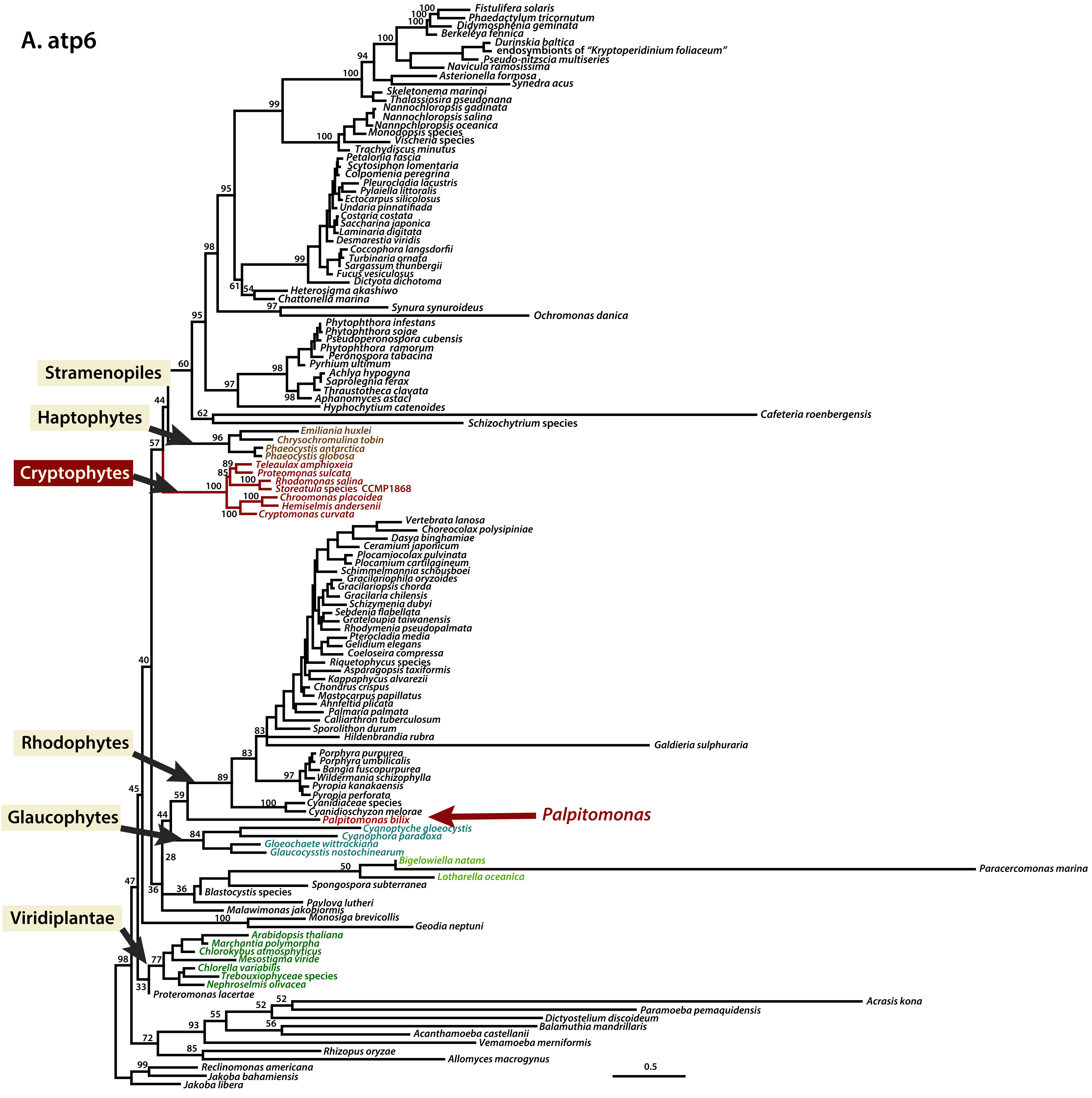

A. atp6
Fistulifera solaris
Phaedactylum tricornutum
Didymosphenia geminata
Berkeleya fennica
Durinskia baltica
endosymbionts of "Kryptoperidinium foliaceum"
Pseudo-nitzscia multiseries
Navicula ramosissima
Asterionella formosa
Synedra acus
Skeletonema marinoi
Thalassiosira pseudonana
Nannochloropsis gadinata
Nannochloropsis salina
Nannochloropsis oceanica
Monodopsis species
Vischeria species
Trachydiscus minutus
Petalonia fascia
Scytosiphon lomentaria
Colpomenia peregrina
Pleurocladia lacustris
Pylaiella littoralis
Ectocarpus silicolosus
Undaria pinnatifiada
Costaria costata
Saccharina japonica
Laminaria digitata
Desmarestia viridis
Coccophora langsdorfii
Turbinaria ornata
Sargassum thunbergii
Fucus vesiculosus
Dictyota dichotoma
Heterosigma akashiwo
Chattonella marina
Synura synuroideus
Ochromonas danica
Phytophthora infestans
Phytophthora sojae
Pseudoperonospora cubensis
Phytophthora ramorum
Peronospora tabacina
Pyrhium ultimum
Achlya hypogyna
Saprolegnia ferax
Thraustotheca clavata
Aphanomyces astaci
Hyphochytium catenoides
Cafeteria roenbergensis
Schizochytrium species
Stramenopiles
Haptophytes
Emiliania huxlei
Chrysochromulina tobin
Phaeocystis antarctica
Phaeocystis globosa
Cryptophytes
Teleaulax amphioxeia
Proteomonas sulcata
Rhodomonas salina
Storeatula species CCMP1868
Chroomonas placoidea
Hemiselmis andersenii
Cryptomonas curvata
Vertebrata lanosa
Choreocolax polysipiniae
Dasya binghamiae
Ceramium japonicum
Plocamiocolax pulvinata
Plocamium cartilagineum
Schimmelmannia schousboei
Gracilariophila oryzoides
Gracilariopsis chorda
Gracilaria chilensis
Schizymenia dubyi
Sebdenia flabellata
Grateloupia taiwanensis
Rhodymenia pseudopalmata
Pterocladia media
Gelidium elegans
Coeloseira compressa
Riquetophycus species
Aspdragopsis taxiformis
Kappaphycus alvarezii
Chondrus crispus
Mastocarpus papillatus
Ahnfeltia plicata
Palmaria palmata
Calliarthron tuberculosum
Sporolithon durum
Hildenbrandia rubra
Galdieria sulphuraria
Rhodophytes
Porphyra purpurea
Porphyra umbilicalis
Bangia fuscopurpurea
Wildermania schizophylla
Pyropia kanakaensis
Pyropia perforata
Cyanidiaceae species
Cyanidioschyzon melorae
Palpitomonas bilix
Palpitomonas
Glaucophytes
Cyanoptyche gloeocystis
Cyanophora paradoxa
Gloeochaete wittrockiana
Glaucocysstis nostochinearum
Bigelowiella natans
Paracercomonas marina
Lotharella oceanica
Spongospora subterranea
Blastocystis species
Pavlova lutheri
Malawimonas jakobiormis
Monosiga brevicollis
Geodia neptuni
Viridiplantae
Arabidopsis thaliana
Marchantia polymorpha
Chlorokybus atmosphyticus
Mesostigma viride
Chlorella variabilis
Trebouxiophyceae species
Nephroselmis olivacea
Proteromonas lacertae
Acrasis kona
Paramoeba pemaquidensis
Dictyostelium discoideum
Balamuthia mandrillaris
Acanthamoeba castellanii
Vemamoeba merniformis
Rhizopus oryzae
Allomyces macrogynus
Reclinomonas americana
Jakoba bahamiensis
Jakoba libera
0.5

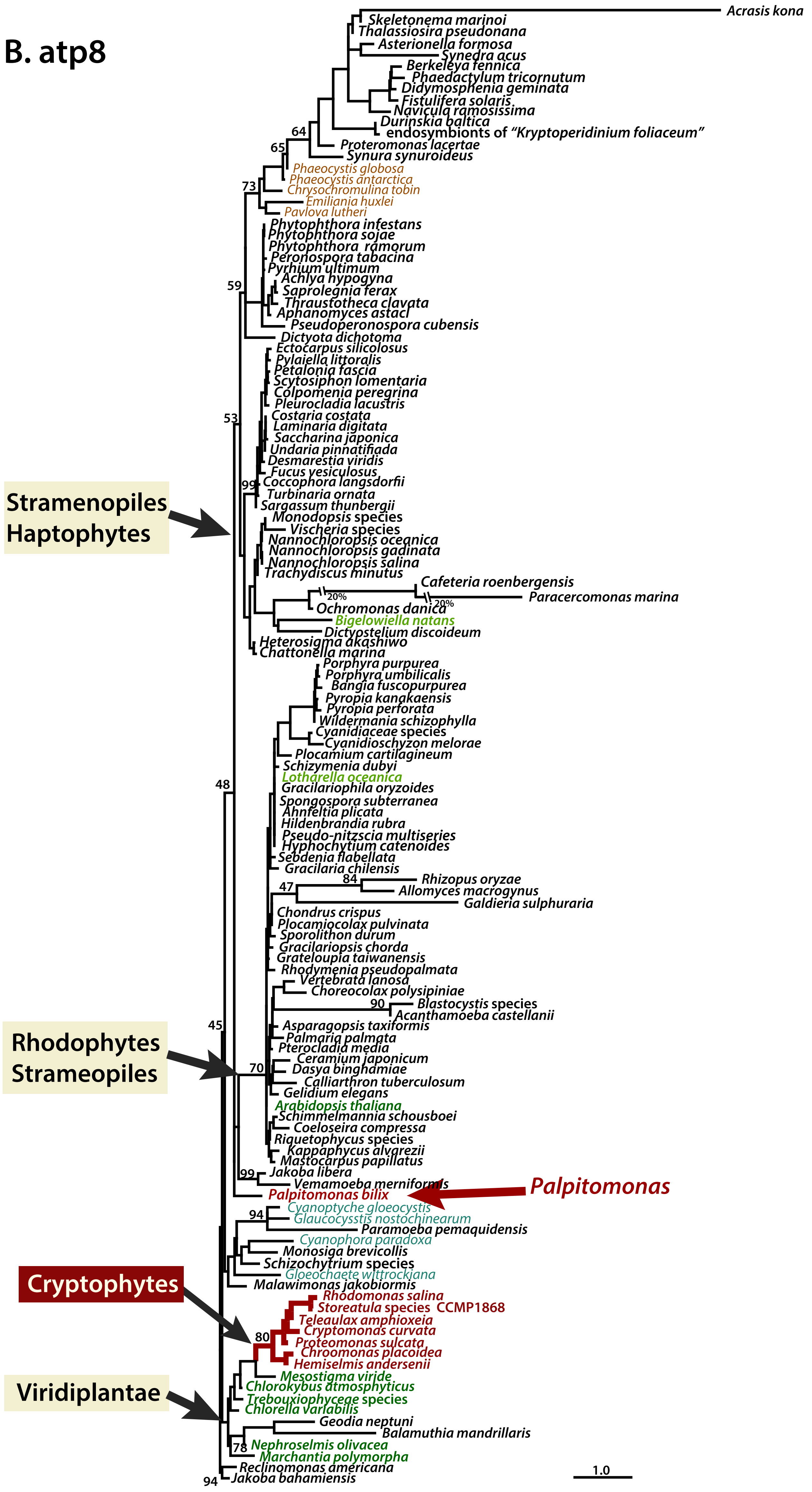
B. atp8
Acrasis kona
Skeletonema marinoi
Thalassiosira pseudonana
Asterionella formosa
Synedra acus
Berkeleya fennica
Phaedactylum tricornutum
Didymosphenia geminata
Fistulifera solaris
Navicula ramosissima
Durinskia baltica
endosymbionts of "Kryptoperidinium foliaceum"
64
65
Proteromonas lacertae
Synura synuroideus
Phaeocystis globosa
Phaeocystis antarctica
73
Chrysochromulina tobin
Emiliania huxlei
Pavlova lutheri
Phytophthora infestans
Phytophthora sojae
Phytophthora ramorum
Peronospora tabacina
Pyrhium ultimum
Achlya hypogyna
59
Saprolegnia ferax
Thraustotheca clavata
Aphanomyces astaci
Pseudoperonospora cubensis
Dictyota dichotoma
Ectocarpus silicolosus
Pylaiella littoralis
Petalonia fascia
Scytosiphon lomentaria
Colpomenia peregrina
Pleurocladia lacustris
Costaria costata
53
Laminaria digitata
Saccharina japonica
Undaria pinnatifiada
Desmarestia viridis
Fucus yesiculosus
99
Coccophora langsdorfii
Turbinaria ornata
Sargassum thunbergii
Stramenopiles
Haptophytes
Monodopsis species
Vischeria species
Nannochloropsis oceanica
Nannochloropsis gadinata
Nannochloropsis salina
Trachydiscus minutus
Cafeteria roenbergensis
20%
Paracercomonas marina
20%
Ochromonas danica
Bigelowiella natans
Dictyostelium discoideum
Heterosigma akashiwo
Chattonella marina
Porphyra purpurea
Porphyra umbilicalis
Bangia fuscopurpurea
Pyropia kanakaensis
Pyropia perforata
Wildermania schizophylla
Cyanidiaceae species
Cyanidioschyzon melorae
Plocamium cartilagineum
Schizymenia dubyi
48
Lotharella oceanica
Gracilariophila oryzoides
Spongospora subterranea
Ahnfeltia plicata
Hildenbrandia rubra
Pseudo-nitzscia multiseries
Hyphochytium catenoides
Sebdenia flabellata
Gracilaria chilensis
84
Rhizopus oryzae
47
Allomyces macrogynus
Galdieria sulphuraria
Chondrus crispus
Plocamiocolax pulvinata
Sporolithon durum
Gracilariopsis chorda
Grateloupia taiwanensis
Rhodymenia pseudopalmata
Vertebrata lanosa
Choreocolax polysipiniae
90
Blastocystis species
Acanthamoeba castellanii
45
Asparagopsis taxiformis
Rhodophytes
Strameopiles
Palmaria palmata
Pterocladia media
Ceramium japonicum
70
Dasya binghamiae
Calliarthron tuberculosum
Gelidium elegans
Arabidopsis thaliana
Schimmelmannia schousboei
Coeloseira compressa
Riquetophycus species
Kappaphycus alvarezii
Mastocarpus papillatus
99
Jakoba libera
Vemamoeba merniformis
Palpitomonas bilix
Palpitomonas
Cyanoptyche gloeocystis
94
Glaucocysstis nostochinearum
Paramoeba pemaquidensis
Cyanophora paradoxa
Monosiga brevicollis
Schizochytrium species
Cryptophytes
Gloeochaete wittrockiana
Malawimonas jakobiormis
Rhodomonas salina
Storeatula species CCMP1868
Teleaulax amphioxeia
Cryptomonas curvata
80
Proteomonas sulcata
Chroomonas placoidea
Hemiselmis andersenii
Mesostigma viride
Chlorokybus atmosphyticus
Viridiplantae
Trebouxiophyceae species
Chlorella variabilis
Geodia neptuni
Balamuthia mandrillaris
78
Nephroselmis olivacea
Marchantia polymorpha
Reclinomonas americana
94
Jakoba bahamiensis
1.0

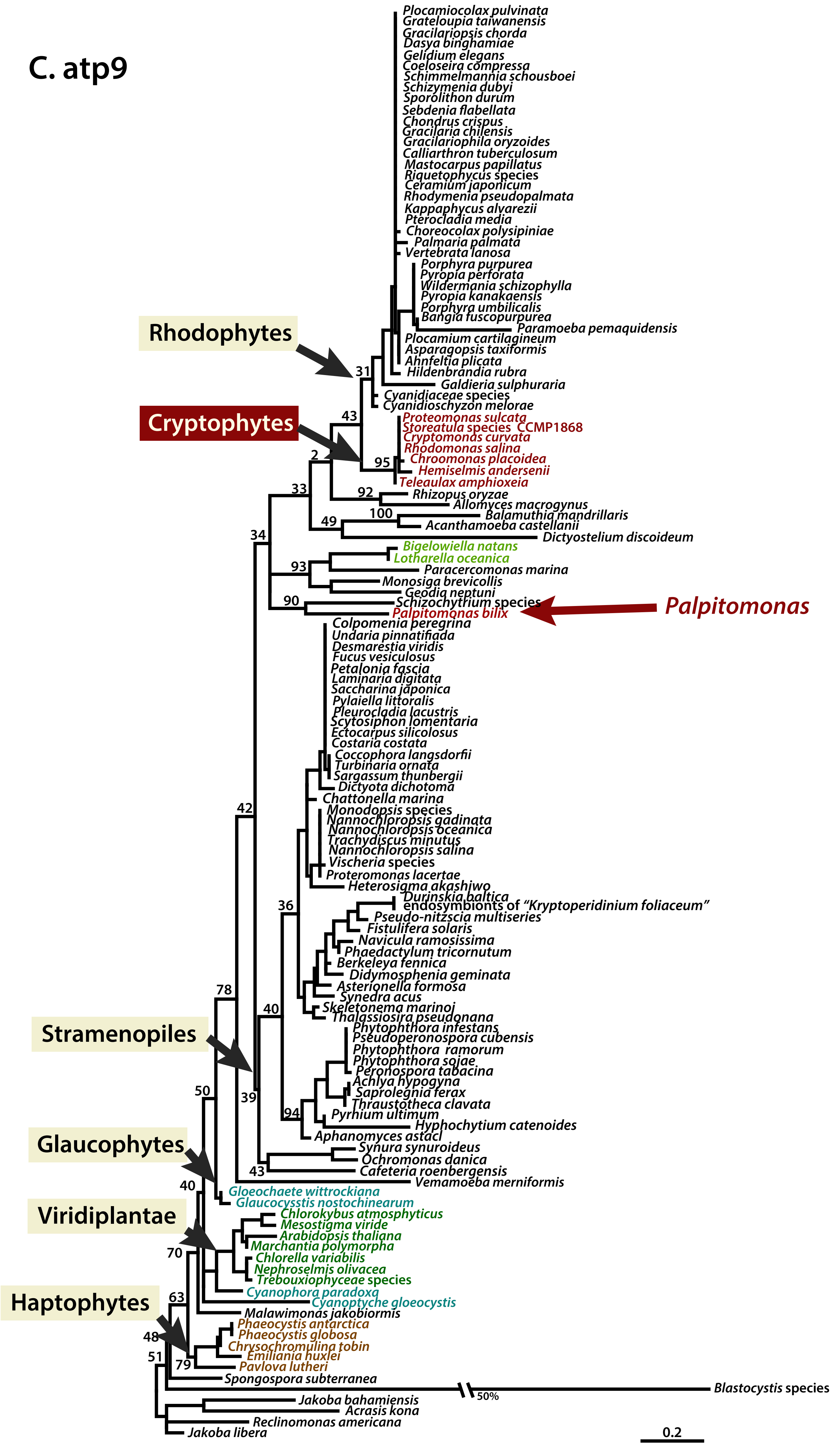
C. atp9
Plocamiocolax pulvinata
Grateloupia taiwanensis
Gracilariopsis chorda
Dasya binghamiae
Gelidium elegans
Coeloseira compressa
Schimmelmannia schousboei
Schizymenia dubyi
Sporolithon durum
Sebdenia flabellata
Chondrus crispus
Gracilaria chilensis
Gracilariophila oryzoides
Calliarthron tuberculosum
Mastocarpus papillatus
Riquetophycus species
Ceramium japonicum
Rhodymenia pseudopalmata
Kappaphycus alvarezii
Pterocladia media
Choreocolax polysipiniae
Palmaria palmata
Vertebrata lanosa
Porphyra purpurea
Pyropia perforata
Wildermania schizophylla
Pyropia kanakaensis
Porphyra umbilicalis
Bangia fuscopurpurea
Paramoeba pemaquidensis
Plocamium cartilagineum
Asparagopsis taxiformis
Ahnfeltia plicata
Hildenbrandia rubra
Galdieria sulphuraria
Cyanidiaceae species
Cyanidioschyzon melorae
Rhodophytes
Cryptophytes
Proteomonas sulcata
Storeatula species CCMP1868
Cryptomonas curvata
Rhodomonas salina
Chroomonas placoidea
Hemiselmis andersenii
Teleaulax amphioxeia
Rhizopus oryzae
Allomyces macrogynus
Balamuthia mandrillaris
Acanthamoeba castellanii
Dictyostelium discoideum
Bigelowiella natans
Lotharella oceanica
Paracercomonas marina
Monosiga brevicollis
Geodia neptuni
Schizochytrium species
Palpitomonas bilix
Palpitomonas
Colpomenia peregrina
Undaria pinnatifiada
Desmarestia viridis
Fucus vesiculosus
Petalonia fascia
Laminaria digitata
Saccharina japonica
Pylaiella littoralis
Pleurocladia lacustris
Scytosiphon lomentaria
Ectocarpus silicolosus
Costaria costata
Coccophora langsdorfii
Turbinaria ornata
Sargassum thunbergii
Dictyota dichotoma
Chattonella marina
Monodopsis species
Nannochloropsis gadinata
Nannochloropsis oceanica
Trachydiscus minutus
Nannochloropsis salina
Vischeria species
Proteromonas lacertae
Heterosigma akashiwo
Durinskia baltica
endosymbionts of "Kryptoperidinium foliaceum"
Pseudo-nitzscia multiseries
Fistulifera solaris
Navicula ramosissima
Phaedactylum tricornutum
Berkeleya fennica
Didymosphenia geminata
Asterionella formosa
Synedra acus
Skeletonema marinoi
Thalassiosira pseudonana
Phytophthora infestans
Pseudoperonospora cubensis
Phytophthora ramorum
Phytophthora sojae
Peronospora tabacina
Achlya hypogyna
Saprolegnia ferax
Thraustotheca clavata
Pyrhium ultimum
Hyphochytium catenoides
Aphanomyces astaci
Synura synuroideus
Ochromonas danica
Cafeteria roenbergensis
Vemamoeba merniformis
Stramenopiles
Glaucophytes
Gloeochaete wittrockiana
Glaucocysstis nostochinearum
Viridiplantae
Chlorokybus atmosphyticus
Mesostigma viride
Arabidopsis thaliana
Marchantia polymorpha
Chlorella variabilis
Nephroselmis olivacea
Trebouxiophyceae species
Cyanophora paradoxa
Cyanoptyche gloeocystis
Malawimonas jakobiormis
Haptophytes
Phaeocystis antarctica
Phaeocystis globosa
Chrysochromulina tobin
Emiliania huxlei
Pavlova lutheri
Spongospora subterranea
Blastocystis species
50%
Jakoba bahamiensis
Acrasis kona
Reclinomonas americana
Jakoba libera
0.2
31
43
2
95
33
92
100
49
34
93
90
42
36
78
40
50
39
94
43
40
70
63
48
51
79

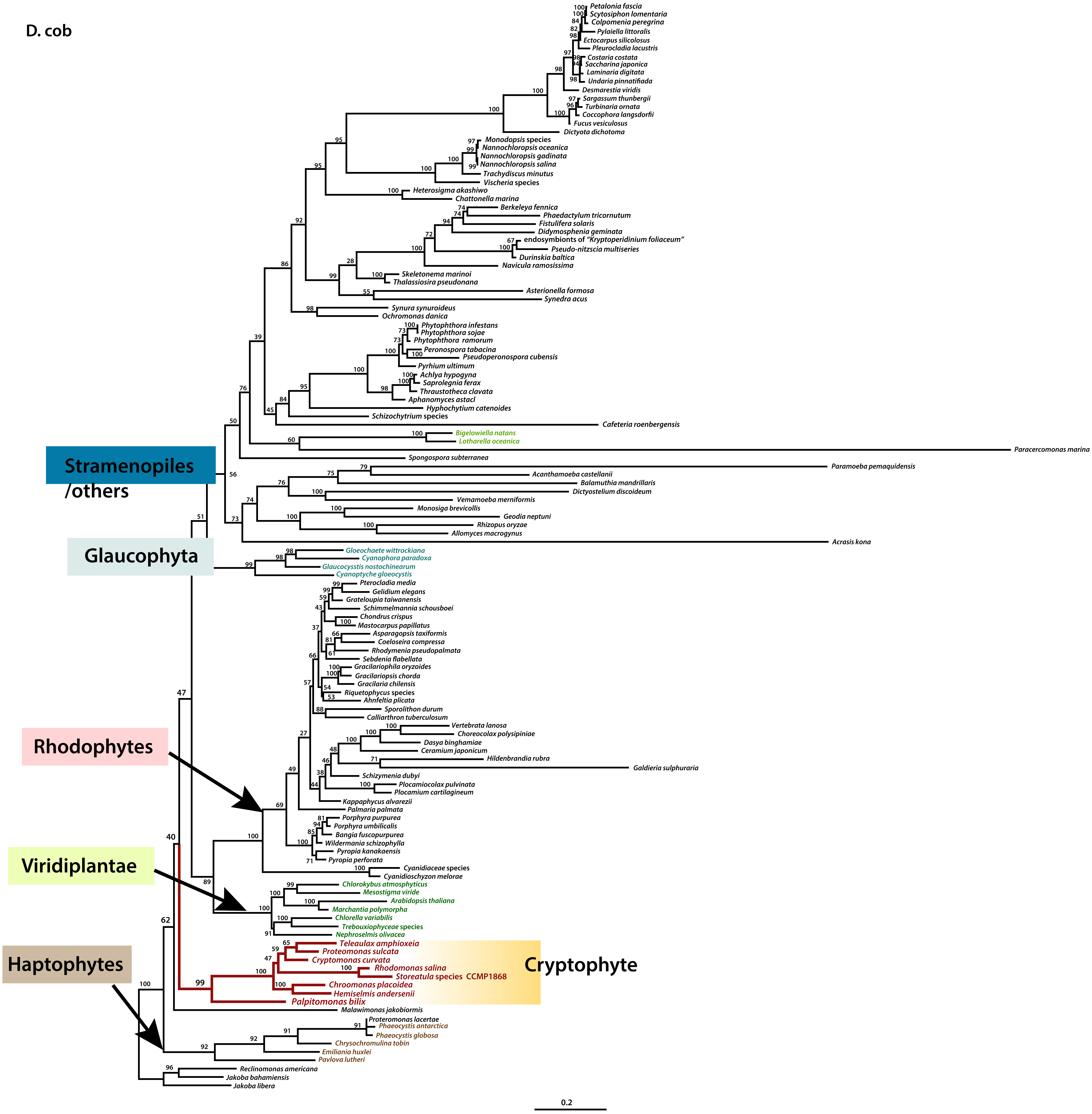
D. cob
Petalonia fascia
Scytosiphon lomentaria
Colpomenia peregrina
Pylaiella littoralis
Ectocarpus silicolosus
Pleurocladia lacustris
Costaria costata
Saccharina japonica
Laminaria digitata
Undaria pinnatifiada
Desmarestia viridis
Sargassum thunbergii
Turbinaria ornata
Coccophora langsdorfii
Fucus vesiculosus
Dictyota dichotoma
Monodopsis species
Nannochloropsis oceanica
Nannochloropsis gadinata
Nannochloropsis salina
Trachydiscus minutus
Vischeria species
Heterosigma akashiwo
Chattonella marina
Berkeleya fennica
Phaedactylum tricornutum
Fistulifera solaris
Didymosphenia geminata
endosymbionts of “Kryptoperidinium foliaceum”
Pseudo-nitzscia multiseries
Durinskia baltica
Navicula ramosissima
Skeletonema marinoi
Thalassiosira pseudonana
Asterionella formosa
Synedra acus
Synura synuroideus
Ochromonas danica
Phytophthora infestans
Phytophthora sojae
Phytophthora ramorum
Peronospora tabacina
Pseudoperonospora cubensis
Pyrhium ultimum
Achlya hypogyna
Saprolegnia ferax
Thraustotheca clavata
Aphanomyces astacl
Hyphochytium catenoides
Schizochytrium species
Cafeteria roenbergensis
Bigelowiella natans
Lotharella oceanica
Paracercomonas marina
Spongospora subterranea
Stramenopiles /others
Paramoeba pemaquidensis
Acanthamoeba castellanii
Balamuthia mandrillaris
Dictyostelium discoideum
Vemamoeba merniformis
Monosiga brevicollis
Geodia neptuni
Rhizopus oryzae
Allomyces macrogynus
Acrasis kona
Glaucophyta
Gloeochaete wittrockiana
Cyanophora paradoxa
Glaucocysstis nostochinearum
Cyanoptyche gloeocystis
Pterocladia media
Gelidium elegans
Grateloupia taiwanensis
Schimmelmannia schousboei
Chondrus crispus
Mastocarpus papillatus
Asparagopsis taxiformis
Coeloseira compressa
Rhodymenia pseudopalmata
Sebdenia flabellata
Gracilariophila oryzoides
Gracilariopsis chorda
Gracilaria chilensis
Riquetophycus species
Ahnfeltia plicata
Sporolithon durum
Calliarthron tuberculosum
Vertebrata lanosa
Choreocolax polysipiniae
Dasya binghamiae
Ceramium japonicum
Hildenbrandia rubra
Galdieria sulphuraria
Schizymenia dubyi
Plocamiocolax pulvinata
Plocamium cartilagineum
Kappaphycus alvarezii
Palmaria palmata
Rhodophytes
Porphyra purpurea
Porphyra umbilicalis
Bangia fuscopurpurea
Wildermania schizophylla
Pyropia kanakaensis
Pyropia perforata
Cyanidiaceae species
Cyanidioschyzon melorae
Viridiplantae
Chlorokybus atmosphyticus
Mesostigma viride
Arabidopsis thaliana
Marchantia polymorpha
Chlorella variabilis
Trebouxiophyceae species
Nephroselmis olivacea
Teleaulax amphioxeia
Proteomonas sulcata
Cryptomonas curvata
Rhodomonas salina
Storeatula species CCMP1868
Chroomonas placoidea
Hemiselmis andersenii
Palpitomonas bilix
Cryptophyte
Haptophytes
Malawimonas jakobiormis
Proteromonas lacertae
Phaeocystis antarctica
Phaeocystis globosa
Chrysochromulina tobin
Emiliania huxlei
Pavlova lutheri
Reclinomonas americana
Jakoba bahamiensis
Jakoba libera
0.2

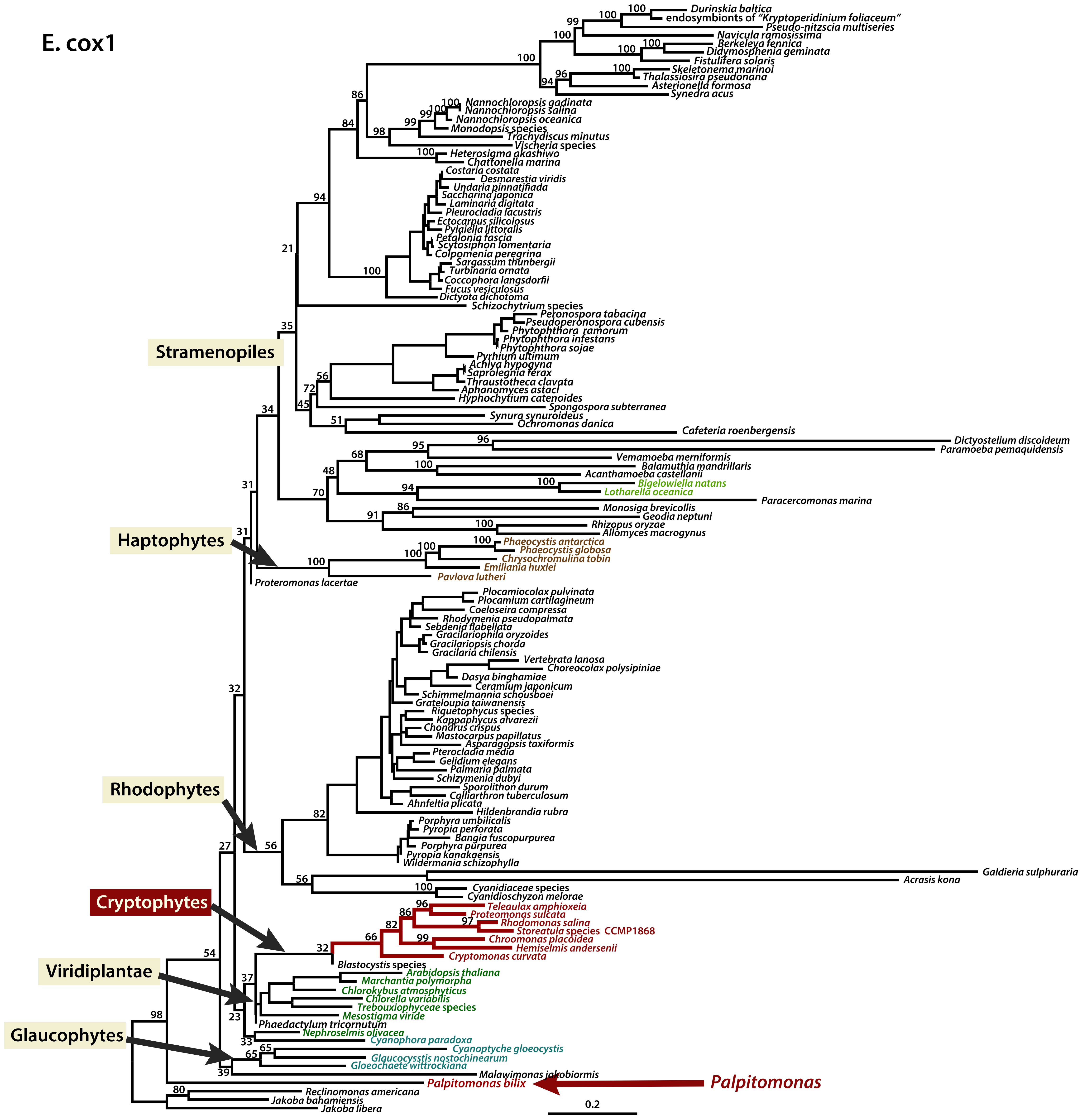
E. cox1
Durinskia baltica
endosymbionts of "Kryptoperidinium foliaceum"
Pseudo-nitzscia multiseries
Navicula ramosissima
Berkeleya fennica
Didymosphenia geminata
Fistulifera solaris
Skeletonema marinoi
Thalassiosira pseudonana
Asterionella formosa
Synedra acus
Nannochloropsis gadinata
Nannochloropsis salina
Nannochloropsis oceanica
Monodopsis species
Trachydiscus minutus
Vischeria species
Heterosigma akashiwo
Chattonella marina
Costaria costata
Desmarestia viridis
Undaria pinnatifiada
Saccharina japonica
Laminaria digitata
Pleurocladia lacustris
Ectocarpus silicolosus
Pylaiella littoralis
Petalonia fascia
Scytosiphon lomentaria
Colpomenia peregrina
Sargassum thunbergii
Turbinaria ornata
Coccophora langsdorfii
Fucus vesiculosus
Dictyota dichotoma
Schizochytrium species
Peronospora tabacina
Pseudoperonospora cubensis
Phytophthora ramorum
Phytophthora infestans
Phytophthora sojae
Pyrhium ultimum
Achlya hypogyna
Saprolegnia ferax
Thraustotheca clavata
Aphanomyces astaci
Hyphochytium catenoides
Spongospora subterranea
Synura synuroideus
Ochromonas danica
Cafeteria roenbergensis
Stramenopiles
Dictyostelium discoideum
Paramoeba pemaquidensis
Vemamoeba merniformis
Balamuthia mandrillaris
Acanthamoeba castellanii
Bigelowiella natans
Lotharella oceanica
Paracercomonas marina
Monosiga brevicollis
Geodia neptuni
Rhizopus oryzae
Allomyces macrogynus
Haptophytes
Phaeocystis antarctica
Phaeocystis globosa
Chrysochromulina tobin
Emiliania huxlei
Pavlova lutheri
Proteromonas lacertae
Plocamiocolax pulvinata
Plocamium cartilagineum
Coeloseira compressa
Rhodymenia pseudopalmata
Sebdenia flabellata
Gracilariophila oryzoides
Gracilariopsis chorda
Gracilaria chilensis
Vertebrata lanosa
Choreocolax polysipiniae
Dasya binghamiae
Ceramium japonicum
Schimmelmannia schousboei
Grateloupia taiwanensis
Riquetophycus species
Kappaphycus alvarezii
Chondrus crispus
Mastocarpus papillatus
Asparagopsis taxiformis
Pterocladia media
Gelidium elegans
Palmaria palmata
Schizymenia dubyi
Sporolithon durum
Calliarthron tuberculosum
Ahnfeltia plicata
Hildenbrandia rubra
Rhodophytes
Porphyra umbilicalis
Pyropia perforata
Bangia fuscopurpurea
Porphyra purpurea
Pyropia kanakaensis
Wildermania schizophylla
Galdieria sulphuraria
Acrasis kona
Cyanidiaceae species
Cyanidioschyzon melorae
Cryptophytes
Teleaulax amphioxeia
Proteomonas sulcata
Rhodomonas salina
Storeatula species CCMP1868
Chroomonas placoidea
Hemiselmis andersenii
Cryptomonas curvata
Blastocystis species
Viridiplantae
Arabidopsis thaliana
Marchantia polymorpha
Chlorokybus atmosphyticus
Chlorella variabilis
Trebouxiophyceae species
Mesostigma viride
Phaedactylum tricornutum
Nephroselmis olivacea
Glaucophytes
Cyanophora paradoxa
Cyanoptyche gloeocystis
Glaucocystis nostochinearum
Gloeochaete wittrockiana
Malawimonas jakobiormis
Palpitomonas bilix
Palpitomonas
Reclinomonas americana
Jakoba bahamiensis
Jakoba libera
0.2

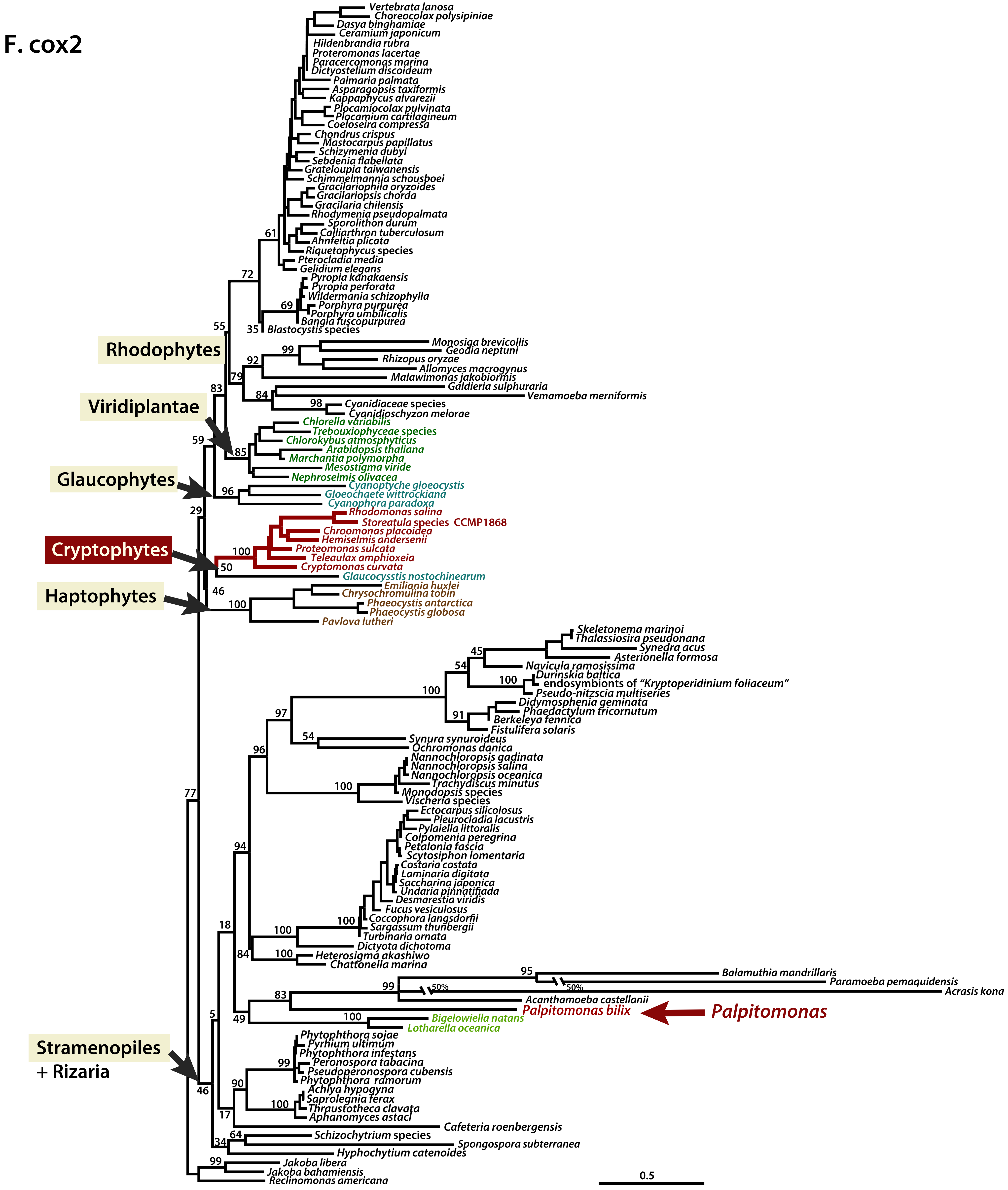
F. cox2
Rhodophytes
Viridiplantae
Glaucophytes
Cryptophytes
Haptophytes
Stramenopiles
+ Rizaria
Palpitomonas
Vertebrata lanosa
Choreocolax polysipiniae
Dasya binghamiae
Ceramium japonicum
Hildenbrandia rubra
Proteromonas lacertae
Paracercomonas marina
Dictyostelium discoideum
Palmaria palmata
Asparagopsis taxiformis
Kappaphycus alvarezii
Plocamiocolax pulvinata
Plocamium cartilagineum
Coeloseira compressa
Chondrus crispus
Mastocarpus papillatus
Schizymenia dubyi
Sebdenia flabellata
Grateloupia taiwanensis
Schimmelmannia schousboei
Gracilariophila oryzoides
Gracilariopsis chorda
Gracilaria chilensis
Rhodymenia pseudopalmata
Sporolithon durum
Calliarthron tuberculosum
Ahnfeltia plicata
Riquetophycus species
Pterocladia media
Gelidium elegans
Pyropia kanakaensis
Pyropia perforata
Wildermania schizophylla
Porphyra purpurea
Porphyra umbilicalis
Bangia fuscopurpurea
Blastocystis species
Monosiga brevicollis
Geodia neptuni
Rhizopus oryzae
Allomyces macrogynus
Malawimonas jakobiormis
Galdieria sulphuraria
Vemamoeba merniformis
Cyanidiaceae species
Cyanidioschyzon melorae
Chlorella variabilis
Trebouxiophyceae species
Chlorokybus atmosphyticus
Arabidopsis thaliana
Marchantia polymorpha
Mesostigma viride
Nephroselmis olivacea
Cyanoptyche gloeocystis
Gloeochaete wittrockiana
Cyanophora paradoxa
Rhodomonas salina
Storeatula species CCMP1868
Chroomonas placoidea
Hemiselmis andersenii
Proteomonas sulcata
Teleaulax amphioxeia
Cryptomonas curvata
Glaucocysstis nostochinearum
Emiliania huxlei
Chrysochromulina tobin
Phaeocystis antarctica
Phaeocystis globosa
Pavlova lutheri
Skeletonema marinoi
Thalassiosira pseudonana
Synedra acus
Asterionella formosa
Navicula ramosissima
Durinskia baltica
endosymbionts of “Kryptoperidinium foliaceum”
Pseudo-nitzscia multiseries
Didymosphenia geminata
Phaedactylum tricornutum
Berkeleya fennica
Fistulifera solaris
Synura synuroideus
Ochromonas danica
Nannochloropsis gadinata
Nannochloropsis salina
Nannochloropsis oceanica
Trachydiscus minutus
Monodopsis species
Vischeria species
Ectocarpus silicolosus
Pleurocladia lacustris
Pylaiella littoralis
Colpomenia peregrina
Petalonia fascia
Scytosiphon lomentaria
Costaria costata
Laminaria digitata
Saccharina japonica
Undaria pinnatifiada
Desmarestia viridis
Fucus vesiculosus
Coccophora langsdorfii
Sargassum thunbergii
Turbinaria ornata
Dictyota dichotoma
Heterosigma akashiwo
Chattonella marina
Balamuthia mandrillaris
Paramoeba pemaquidensis
Acrasis kona
Acanthamoeba castellanii
Palpitomonas bilix
Bigelowiella natans
Lotharella oceanica
Phytophthora sojae
Pyrhium ultimum
Phytophthora infestans
Peronospora tabacina
Pseudoperonospora cubensis
Phytophthora ramorum
Achlya hypogyna
Saprolegnia ferax
Thraustotheca clavata
Aphanomyces astaci
Cafeteria roenbergensis
Schizochytrium species
Spongospora subterranea
Hyphochytium catenoides
Jakoba libera
Jakoba bahamiensis
Reclinomonas americana
50%
0.5

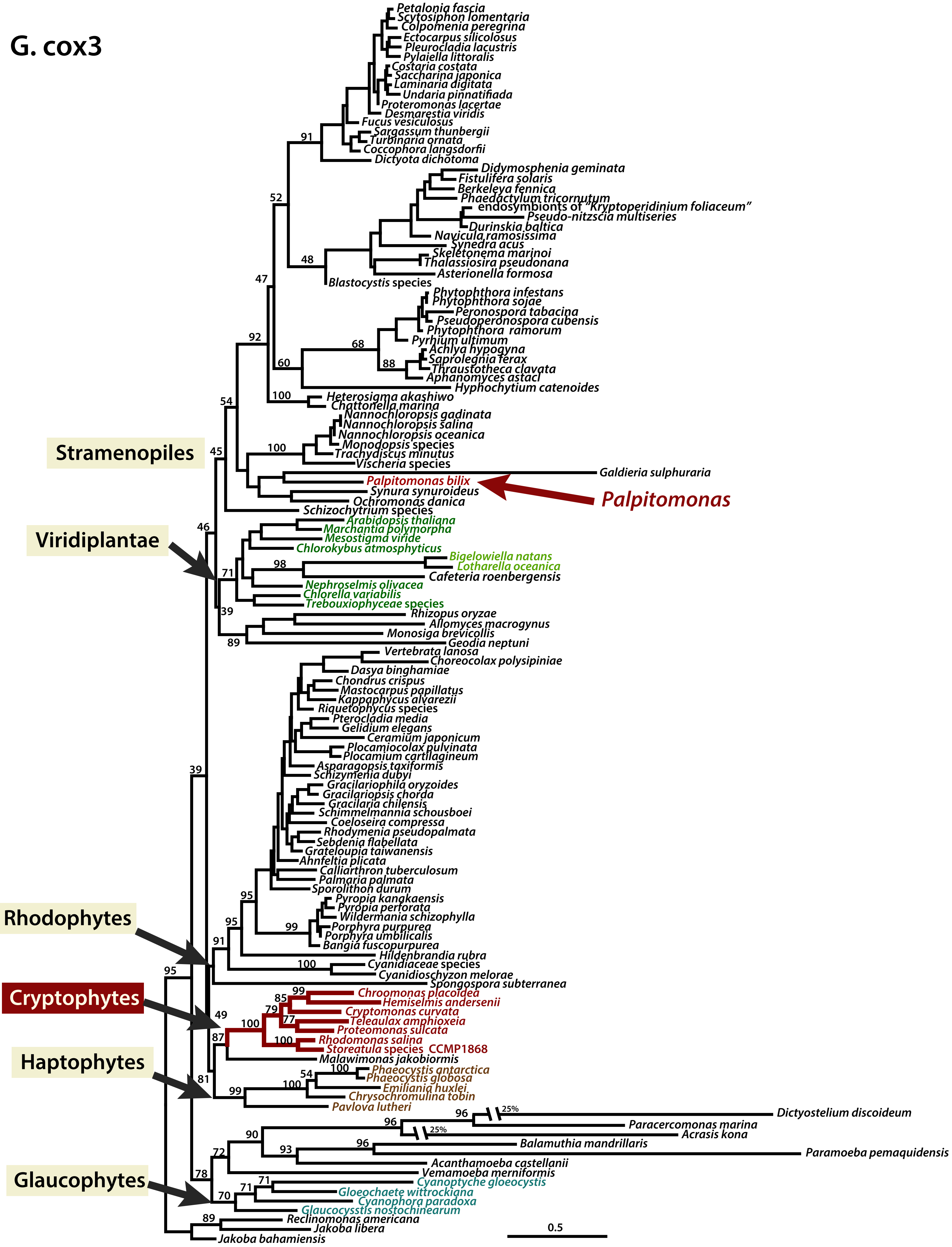
G. cox3
Petalonia fascia
Scytosiphon lomentaria
Colpomenia peregrina
Ectocarpus silicolosus
Pleurocladia lacustris
Pylaiella littoralis
Costaria costata
Saccharina japonica
Laminaria digitata
Undaria pinnatifiada
Proteromonas lacertae
Desmarestia viridis
Fucus vesiculosus
Sargassum thunbergii
Turbinaria ornata
Coccophora langsdorfii
Dictyota dichotoma
Didymosphenia geminata
Fistulifera solaris
Berkeleya fennica
Phaedactylum tricornutum
endosymbionts of "Kryptoperidinium foliaceum"
Pseudo-nitzscia multiseries
Durinskia baltica
Navicula ramosissima
Synedra acus
Skeletonema marinoi
Thalassiosira pseudonana
Asterionella formosa
Blastocystis species
Phytophthora infestans
Phytophthora sojae
Peronospora tabacina
Pseudoperonospora cubensis
Phytophthora ramorum
Pyrhium ultimum
Achlya hypogyna
Saprolegnia ferax
Thraustotheca clavata
Aphanomyces astaci
Hyphochytium catenoides
Heterosigma akashiwo
Chattonella marina
Nannochloropsis gadinata
Nannochloropsis salina
Nannochloropsis oceanica
Monodopsis species
Trachydiscus minutus
Vischeria species
Galdieria sulphuraria
Palpitomonas bilix
Synura synuroideus
Ochromonas danica
Schizochytrium species
Palpitomonas
Stramenopiles
Viridiplantae
Arabidopsis thaliana
Marchantia polymorpha
Mesostigma viride
Chlorokybus atmosphyticus
Bigelowiella natans
Lotharella oceanica
Cafeteria roenbergensis
Nephroselmis olivacea
Chlorella variabilis
Trebouxiophyceae species
Rhizopus oryzae
Allomyces macrogynus
Monosiga brevicollis
Geodia neptuni
Vertebrata lanosa
Choreocolax polysipiniae
Dasya binghamiae
Chondrus crispus
Mastocarpus papillatus
Kappaphycus alvarezii
Riquetophycus species
Pterocladia media
Gelidium elegans
Ceramium japonicum
Plocamiocolax pulvinata
Plocamium cartilagineum
Asparagopsis taxiformis
Schizymenia dubyi
Gracilariophila oryzoides
Gracilariopsis chorda
Gracilaria chilensis
Schimmelmannia schousboei
Coeloseira compressa
Rhodymenia pseudopalmata
Sebdenia flabellata
Grateloupia taiwanensis
Ahnfeltia plicata
Calliarthron tuberculosum
Palmaria palmata
Sporolithon durum
Pyropia kanakaensis
Pyropia perforata
Wildermania schizophylla
Porphyra purpurea
Porphyra umbilicalis
Bangia fuscopurpurea
Hildenbrandia rubra
Cyanidiaceae species
Cyanidioschyzon melorae
Spongospora subterranea
Rhodophytes
Cryptophytes
Chroomonas placoidea
Hemiselmis andersenii
Cryptomonas curvata
Teleaulax amphioxeia
Proteomonas sulcata
Rhodomonas salina
Storeatula species CCMP1868
Malawimonas jakobiormis
Haptophytes
Phaeocystis antarctica
Phaeocystis globosa
Emiliania huxlei
Chrysochromulina tobin
Pavlova lutheri
Dictyostelium discoideum
Paracercomonas marina
Acrasis kona
Balamuthia mandrillaris
Paramoeba pemaquidensis
Acanthamoeba castellanii
Vemamoeba merniformis
Glaucophytes
Cyanoptyche gloeocystis
Gloeochaete wittrockiana
Cyanophora paradoxa
Glaucocysstis nostochinearum
Reclinomonas americana
Jakoba libera
Jakoba bahamiensis
25%
0.5

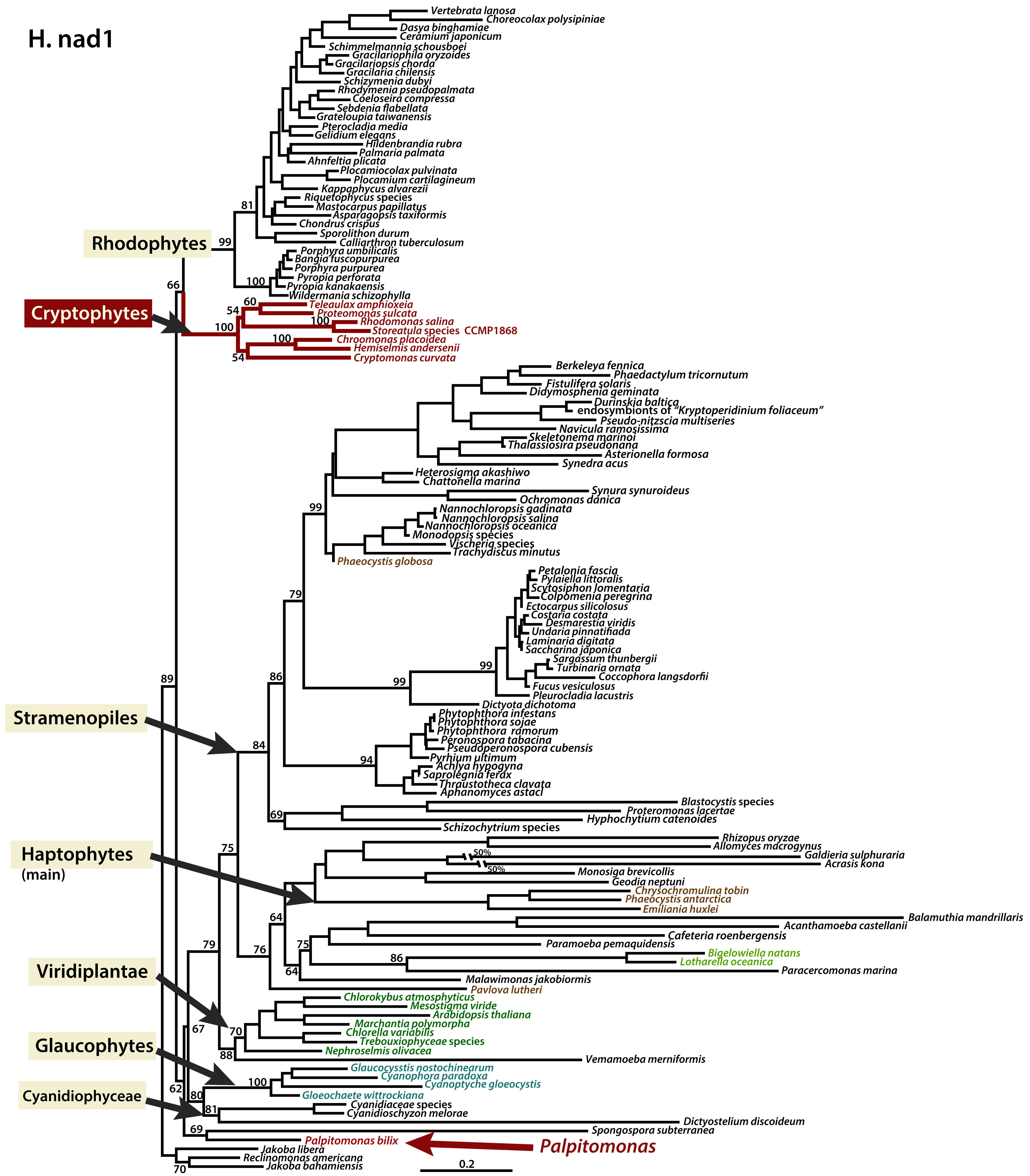
H. nad1
Vertebrata lanosa
Choreocolax polysipiniae
Dasya binghamiae
Ceramium japonicum
Schimmelmannia schousboei
Gracilariophila oryzoides
Gracilariopsis chorda
Gracilaria chilensis
Schizymenia dubyi
Rhodymenia pseudopalmata
Coeloseira compressa
Sebdenia flabellata
Grateloupia taiwanensis
Pterocladia media
Gelidium elegans
Hildenbrandia rubra
Palmaria palmata
Ahnfeltia plicata
Plocamiocolax pulvinata
Plocamium cartilagineum
Kappaphycus alvarezii
Riquetophycus species
Mastocarpus papillatus
Asparagopsis taxiformis
Chondrus crispus
Sporolithon durum
Calliarthron tuberculosum
Porphyra umbilicalis
Bangia fuscopurpurea
Porphyra purpurea
Pyropia perforata
Pyropia kanakaensis
Wildermania schizophylla
81
99
100
Rhodophytes
66
Cryptophytes
60
54
100
100
100
54
Teleaulax amphioxeia
Proteomonas sulcata
Rhodomonas salina
Storeatula species CCMP1868
Chroomonas placoidea
Hemiselmis andersenii
Cryptomonas curvata
Berkeleya fennica
Phaedactylum tricornutum
Fistulifera solaris
Didymosphenia geminata
Durinskia baltica
endosymbionts of "Kryptoperidinium foliaceum"
Pseudo-nitzscia multiseries
Navicula ramosissima
Skeletonema marinoi
Thalassiosira pseudonana
Asterionella formosa
Synedra acus
Heterosigma akashiwo
Chattonella marina
Synura synuroideus
Ochromonas danica
99
Nannochloropsis gadinata
Nannochloropsis salina
Nannochloropsis oceanica
Monodopsis species
Vischeria species
Trachydiscus minutus
Phaeocystis globosa
Petalonia fascia
Pylaiella littoralis
Scytosiphon lomentaria
Colpomenia peregrina
Ectocarpus silicolosus
Costaria costata
Desmarestia viridis
Undaria pinnatifiada
Laminaria digitata
Saccharina japonica
Sargassum thunbergii
Turbinaria ornata
Coccophora langsdorfii
Fucus vesiculosus
Pleurocladia lacustris
Dictyota dichotoma
79
99
99
86
89
Stramenopiles
84
Phytophthora infestans
Phytophthora sojae
Phytophthora ramorum
Peronospora tabacina
Pseudoperonospora cubensis
Pyrhium ultimum
Achlya hypogyna
Saprolegnia ferax
Thraustotheca clavata
Aphanomyces astaci
94
Blastocystis species
Proteromonas lacertae
Hyphochytium catenoides
Schizochytrium species
69
Haptophytes
(main)
75
Rhizopus oryzae
Allomyces macrogynus
50%
Galdieria sulphuraria
50%
Acrasis kona
Monosiga brevicollis
Geodia neptuni
Chrysochromulina tobin
Phaeocystis antarctica
Emiliania huxlei
64
Balamuthia mandrillaris
Acanthamoeba castellanii
Cafeteria roenbergensis
Paramoeba pemaquidensis
75
79
76
86
Bigelowiella natans
Lotharella oceanica
64
Paracercomonas marina
Malawimonas jakobiormis
Pavlova lutheri
Viridiplantae
Chlorokybus atmosphyticus
Mesostigma viride
Arabidopsis thaliana
Marchantia polymorpha
67
70
Chlorella variabilis
Trebouxiophyceae species
Nephroselmis olivacea
88
Vemamoeba merniformis
Glaucophytes
Glaucocysstis nostochinearum
Cyanophora paradoxa
100
Cyanoptyche gloeocystis
62
Gloeochaete wittrockiana
80
Cyanidiophyceae
Cyanidiaceae species
81
Cyanidioschyzon melorae
Dictyostelium discoideum
69
Spongospora subterranea
Palpitomonas bilix
Palpitomonas
Jakoba libera
Reclinomonas americana
70
Jakoba bahamiensis
0.2

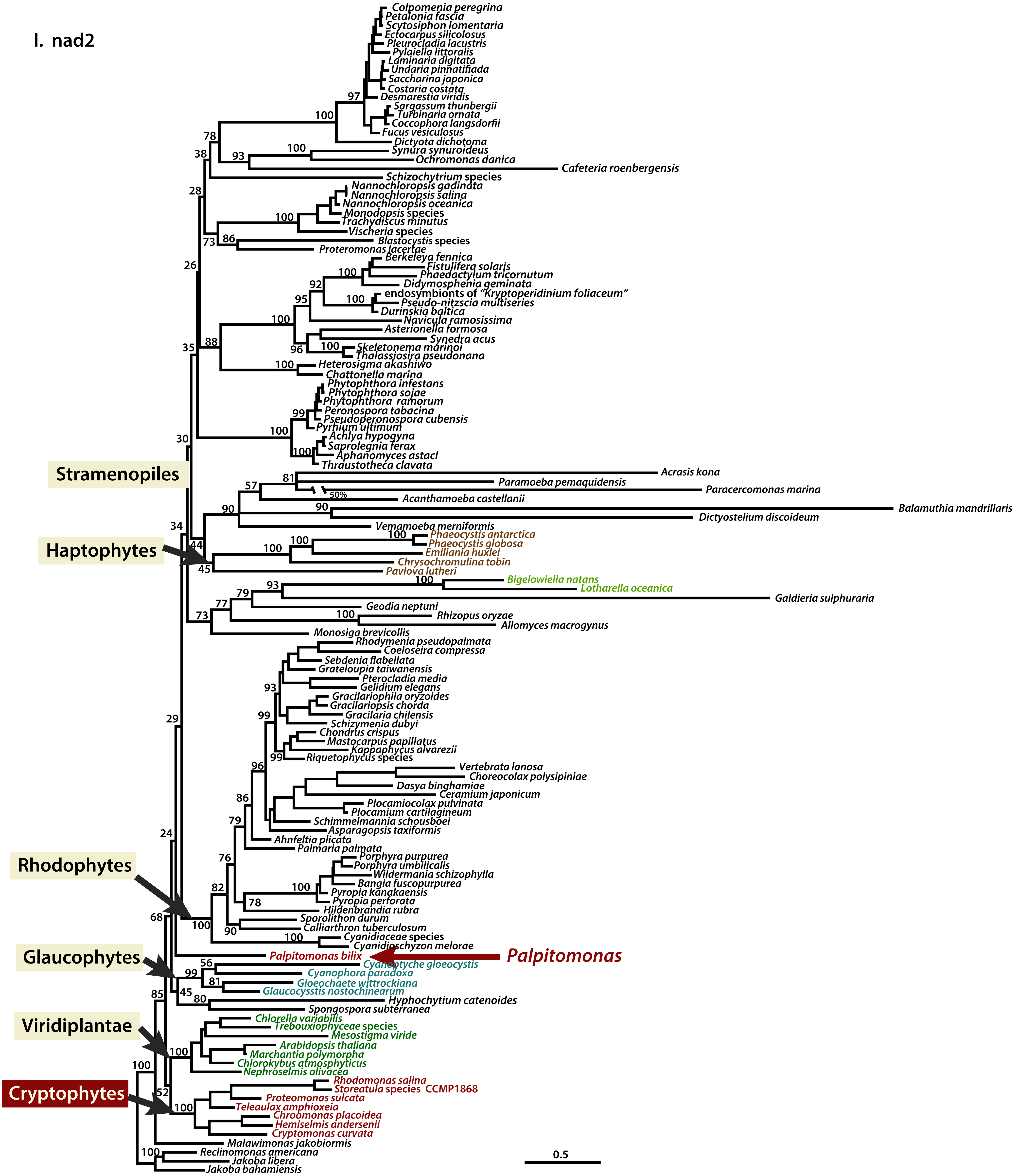
I. nad2
Colpomenia peregrina
Petalonia fascia
Scytosiphon lomentaria
Ectocarpus silicolosus
Pleurocladia lacustris
Pylaiella littoralis
Laminaria digitata
Undaria pinnatifiada
Saccharina japonica
Costaria costata
Desmarestia viridis
Sargassum thunbergii
Turbinaria ornata
Coccophora langsdorfii
Fucus vesiculosus
Dictyota dichotoma
Synura synuroideus
Ochromonas danica
Cafeteria roenbergensis
Schizochytrium species
Nannochloropsis gadinata
Nannochloropsis salina
Nannochloropsis oceanica
Monodopsis species
Trachydiscus minutus
Vischeria species
Blastocystis species
Proteromonas lacertae
Berkeleya fennica
Fistulifera solaris
Phaedactylum tricornutum
Didymosphenia geminata
endosymbionts of "Kryptoperidinium foliaceum"
Pseudo-nitzscia multiseries
Durinskia baltica
Navicula ramosissima
Asterionella formosa
Synedra acus
Skeletonema marinoi
Thalassiosira pseudonana
Heterosigma akashiwo
Chattonella marina
Phytophthora infestans
Phytophthora sojae
Phytophthora ramorum
Peronospora tabacina
Pseudoperonospora cubensis
Pyrhium ultimum
Achlya hypogyna
Saprolegnia ferax
Aphanomyces astacl
Thraustotheca clavata
Stramenopiles
Acrasis kona
Paramoeba pemaquidensis
Paracercomonas marina
50%
Acanthamoeba castellanii
Balamuthia mandrillaris
Dictyostelium discoideum
Vemamoeba merniformis
Haptophytes
Phaeocystis antarctica
Phaeocystis globosa
Emiliania huxlei
Chrysochromulina tobin
Pavlova lutheri
Bigelowiella natans
Lotharella oceanica
Galdieria sulphuraria
Geodia neptuni
Rhizopus oryzae
Allomyces macrogynus
Monosiga brevicollis
Rhodymenia pseudopalmata
Coeloseira compressa
Sebdenia flabellata
Grateloupia taiwanensis
Pterocladia media
Gelidium elegans
Gracilariophila oryzoides
Gracilariopsis chorda
Gracilaria chilensis
Schizymenia dubyi
Chondrus crispus
Mastocarpus papillatus
Kappaphycus alvarezii
Riquetophycus species
Vertebrata lanosa
Choreocolax polysipiniae
Dasya binghamiae
Ceramium japonicum
Plocamiocolax pulvinata
Plocamium cartilagineum
Schimmelmannia schousboei
Asparagopsis taxiformis
Ahnfeltia plicata
Palmaria palmata
Rhodophytes
Porphyra purpurea
Porphyra umbilicalis
Wildermania schizophylla
Bangia fuscopurpurea
Pyropia kanakaensis
Pyropia perforata
Hildenbrandia rubra
Sporolithon durum
Calliarthron tuberculosum
Cyanidiaceae species
Cyanidioschyzon melorae
Palpitomonas bilix
Palpitomonas
Glaucophytes
Cyanoptyche gloeocystis
Cyanophora paradoxa
Gloeochaete wittrockiana
Glaucocysstis nostochinearum
Hyphochytium catenoides
Spongospora subterranea
Viridiplantae
Chlorella variabilis
Trebouxiophyceae species
Mesostigma viride
Arabidopsis thaliana
Marchantia polymorpha
Chlorokybus atmosphyticus
Nephroselmis olivacea
Cryptophytes
Rhodomonas salina
Storeatula species CCMP1868
Proteomonas sulcata
Teleaulax amphioxeia
Chroomonas placoidea
Hemiselmis andersenii
Cryptomonas curvata
Malawimonas jakobiormis
Reclinomonas americana
Jakoba libera
Jakoba bahamiensis
0.5

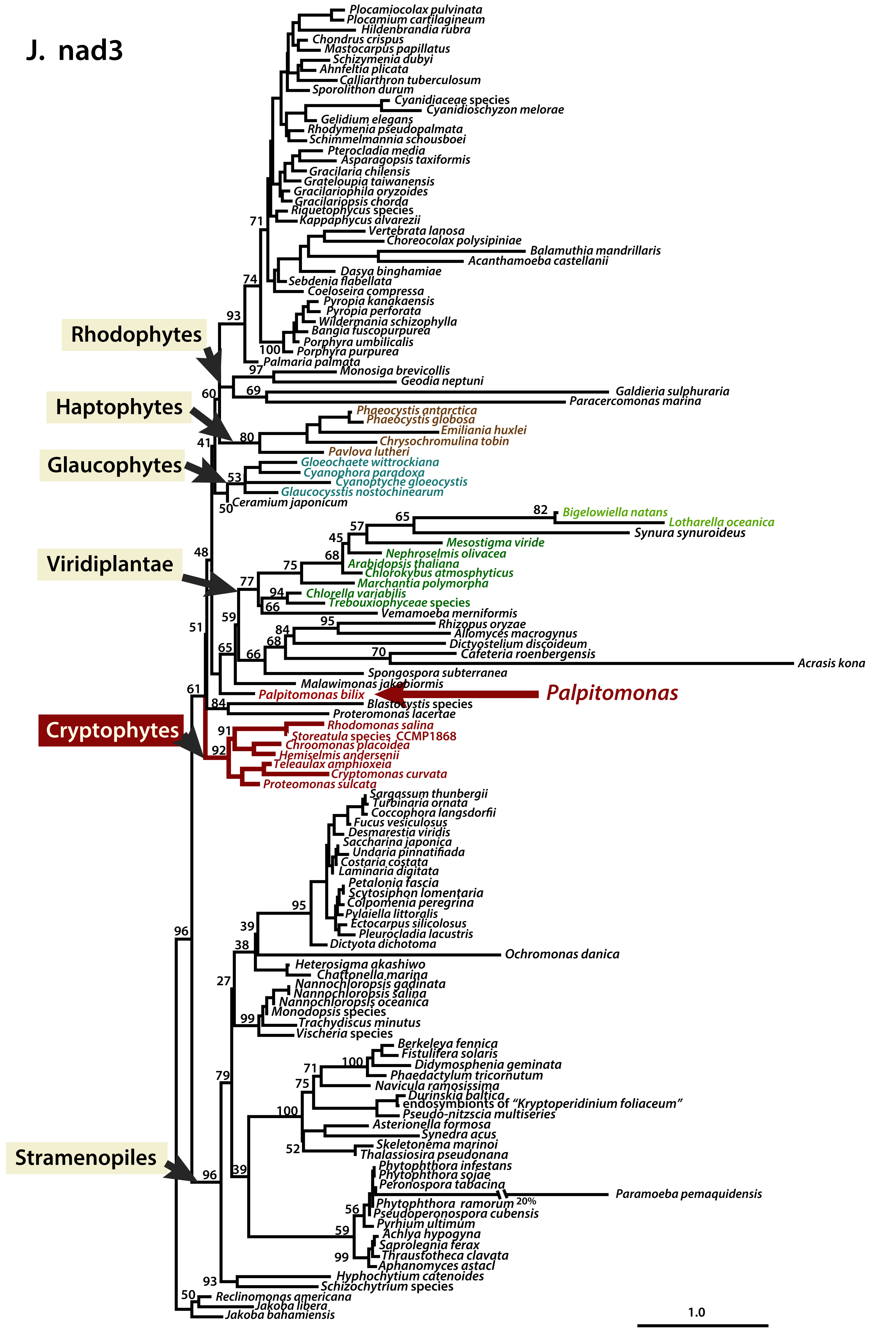

J. nad3
Plocamiocolax pulvinata
Plocamium cartilagineum
Hildenbrandia rubra
Chondrus crispus
Mastocarpus papillatus
Schizymenia dubyi
Ahnfeltia plicata
Calliarthron tuberculosum
Sporolithon durum
Cyanidiaceae species
Cyanidioschyzon melorae
Gelidium elegans
Rhodymenia pseudopalmata
Schimmelmannia schousboei
Pterocladia media
Asparagopsis taxiformis
Gracilaria chilensis
Grateloupia taiwanensis
Gracilariophila oryzoides
Gracilariopsis chorda
Riquetophycus species
Kappaphycus alvarezii
Vertebrata lanosa
Choreocolax polysipiniae
Balamuthia mandrillaris
Acanthamoeba castellanii
Dasya binghamiae
Sebdenia flabellata
Coeloseira compressa
Pyropia kanakaensis
Pyropia perforata
Wildermania schizophylla
Bangia fuscopurpurea
Porphyra umbilicalis
Porphyra purpurea
Palmaria palmata
Monosiga brevicollis
Geodia neptuni
Galdieria sulphuraria
Paracercomonas marina
Phaeocystis antarctica
Phaeocystis globosa
Emiliania huxlei
Chrysochromulina tobin
Pavlova lutheri
Gloeochaete wittrockiana
Cyanophora paradoxa
Cyanoptyche gloeocystis
Glaucocysstis nostochinearum
Ceramium japonicum
Bigelowiella natans
Lotharella oceanica
Synura synuroideus
Mesostigma viride
Nephroselmis olivacea
Arabidopsis thaliana
Chlorokybus atmosphyticus
Marchantia polymorpha
Chlorella variabilis
Trebouxiophyceae species
Vemamoeba merniformis
Rhizopus oryzae
Allomyces macrogynus
Dictyostelium discoideum
Cafeteria roenbergensis
Acrasis kona
Spongospora subterranea
Malawimonas jakobiormis
Palpitomonas bilix
Palpitomonas
Blastocystis species
Proteromonas lacertae
Rhodomonas salina
Storeatula species CCMP1868
Chroomonas placoidea
Hemiselmis andersenii
Teleaulax amphioxeia
Cryptomonas curvata
Proteomonas sulcata
Sargassum thunbergii
Turbinaria ornata
Coccophora langsdorfii
Fucus vesiculosus
Desmarestia viridis
Saccharina japonica
Undaria pinnatifiada
Costaria costata
Laminaria digitata
Petalonia fascia
Scytosiphon lomentaria
Colpomenia peregrina
Pylaiella littoralis
Ectocarpus silicolosus
Pleurocladia lacustris
Dictyota dichotoma
Ochromonas danica
Heterosigma akashiwo
Chattonella marina
Nannochloropsis gadinata
Nannochloropsis salina
Nannochloropsis oceanica
Monodopsis species
Trachydiscus minutus
Vischeria species
Berkeleya fennica
Fistulifera solaris
Didymosphenia geminata
Phaedactylum tricornutum
Navicula ramosissima
Durinskia baltica
endosymbionts of "Kryptoperidinium foliaceum"
Pseudo-nitzscia multiseries
Asterionella formosa
Synedra acus
Skeletonema marinoi
Thalassiosira pseudonana
Phytophthora infestans
Phytophthora sojae
Peronospora tabacina
Paramoeba pemaquidensis
20%
Phytophthora ramorum
Pseudoperonospora cubensis
Pyrhium ultimum
Achlya hypogyna
Saprolegnia ferax
Thraustotheca clavata
Aphanomyces astaci
Hyphochytium catenoides
Schizochytrium species
Reclinomonas americana
Jakoba libera
Jakoba bahamiensis
Rhodophytes
Haptophytes
Glaucophytes
Viridiplantae
Cryptophytes
Stramenopiles
71
74
93
100
97
60
69
80
41
53
50
82
65
57
45
68
75
48
77
94
66
59
95
84
68
51
65
66
70
61
84
91
92
95
39
38
96
27
99
79
71
100
75
100
52
39
56
59
99
96
93
50
1.0

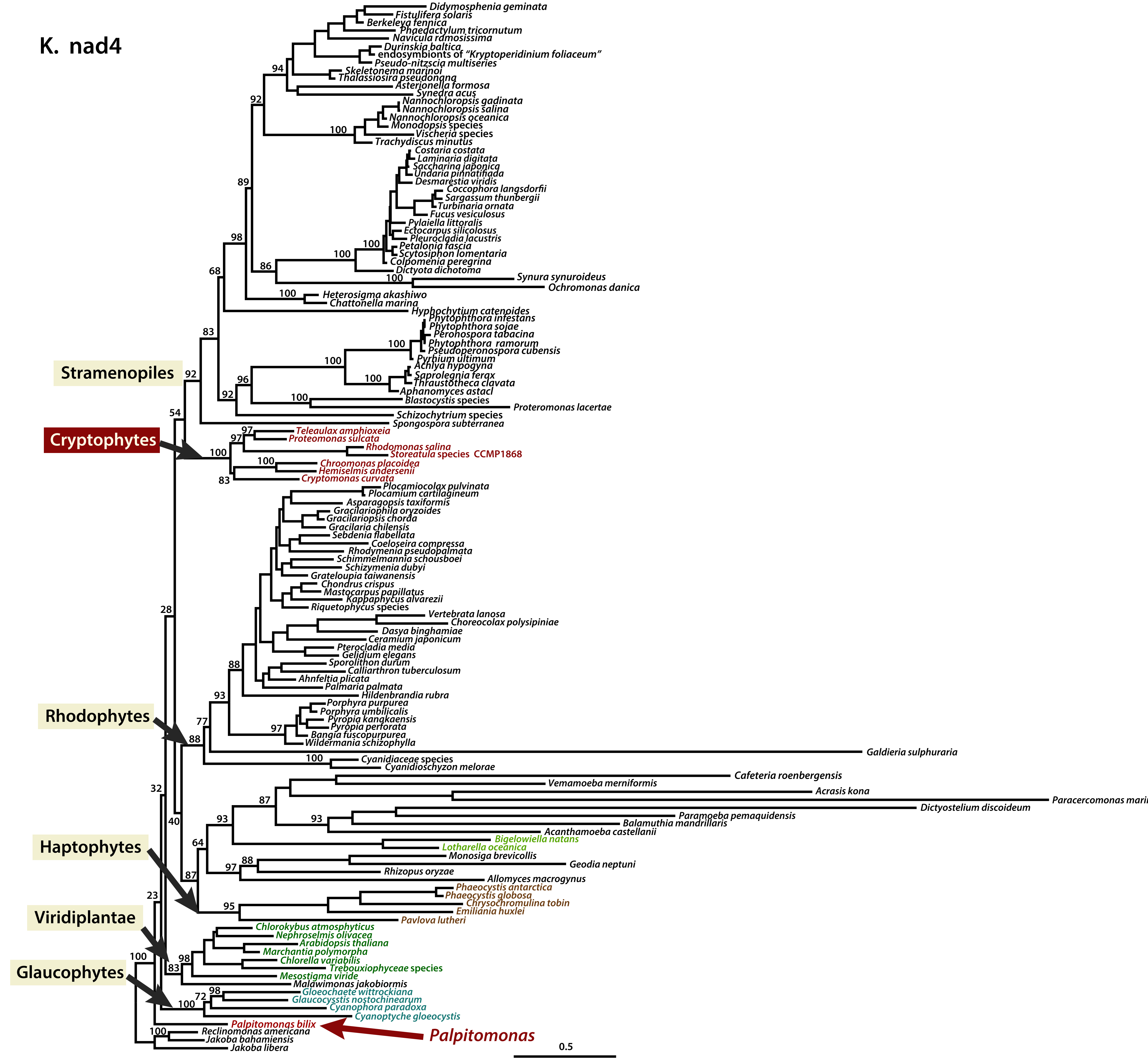
K. nad4
Didymosphenia geminata
Fistulifera solaris
Berkeleya fennica
Phaeodactylum tricornutum
Navicula ramosissima
Durinskia baltica
endosymbionts of "Kryptoperidinium foliaceum"
Pseudo-nitzscia multiseries
Skeletonema marinoi
Thalassiosira pseudonana
Asterionella formosa
Synedra acus
Nannochloropsis gadinata
Nannochloropsis salina
Nannochloropsis oceanica
Monodopsis species
Vischeria species
Trachydiscus minutus
Costaria costata
Laminaria digitata
Saccharina japonica
Undaria pinnatifiada
Desmarestia viridis
Coccophora langsdorfii
Sargassum thunbergii
Turbinaria ornata
Fucus vesiculosus
Pylaiella littoralis
Ectocarpus silicolosus
Pleurocladia lacustris
Petalonia fascia
Scytosiphon lomentaria
Colpomenia peregrina
Dictyota dichotoma
Synura synuroideus
Ochromonas danica
Heterosigma akashiwo
Chattonella marina
Hyphochytium catenoides
Phytophthora infestans
Phytophthora sojae
Perohospora tabacina
Phytophthora ramorum
Pseudoperonospora cubensis
Pyrhium ultimum
Achlya hypogyna
Saprolegnia ferax
Thraustotheca clavata
Aphanomyces astaci
Blastocystis species
Proteromonas lacertae
Schizochytrium species
Spongospora subterranea
Stramenopiles
Cryptophytes
Teleaulax amphioxeia
Proteomonas sulcata
Rhodomonas salina
Storeatula species CCMP1868
Chroomonas placoidea
Hemiselmis andersenii
Cryptomonas curvata
Plocamiocolax pulvinata
Plocamium cartilagineum
Asparagopsis taxiformis
Gracilariophila oryzoides
Gracilariopsis chorda
Gracilaria chilensis
Sebdenia flabellata
Coeloseira compressa
Rhodymenia pseudopalmata
Schimmelmannia schousboei
Schizymenia dubyi
Grateloupia taiwanensis
Chondrus crispus
Mastocarpus papillatus
Kappaphycus alvarezii
Riquetophycus species
Vertebrata lanosa
Choreocolax polysipiniae
Dasya binghamiae
Ceramium japonicum
Pterocladia media
Gelidium elegans
Sporolithon durum
Calliarthron tuberculosum
Ahnfeltia plicata
Palmaria palmata
Hildenbrandia rubra
Porphyra purpurea
Porphyra umbilicalis
Pyropia kanakaensis
Pyropia perforata
Bangia fuscopurpurea
Wildermania schizophylla
Galdieria sulphuraria
Cyanidiaceae species
Cyanidioschyzon melorae
Rhodophytes
Cafeteria roenbergensis
Vemamoeba merniformis
Acrasis kona
Paracercomonas mari
Dictyostelium discoideum
Paramoeba pemaquidensis
Balamuthia mandrillaris
Acanthamoeba castellanii
Bigelowiella natans
Lotharella oceanica
Monosiga brevicollis
Geodia neptuni
Rhizopus oryzae
Allomyces macrogynus
Haptophytes
Phaeocystis antarctica
Phaeocystis globosa
Chrysochromulina tobin
Emiliania huxlei
Pavlova lutheri
Viridiplantae
Chlorokybus atmosphyticus
Nephroselmis olivacea
Arabidopsis thaliana
Marchantia polymorpha
Chlorella variabilis
Trebouxiophyceae species
Mesostigma viride
Malawimonas jakobiormis
Glaucophytes
Gloeochaete wittrockiana
Glaucocysstis nostochinearum
Cyanophora paradoxa
Cyanoptyche gloeocystis
Palpitomonas bilix
Palpitomonas
Reclinomonas americana
Jakoba bahamiensis
Jakoba libera
0.5

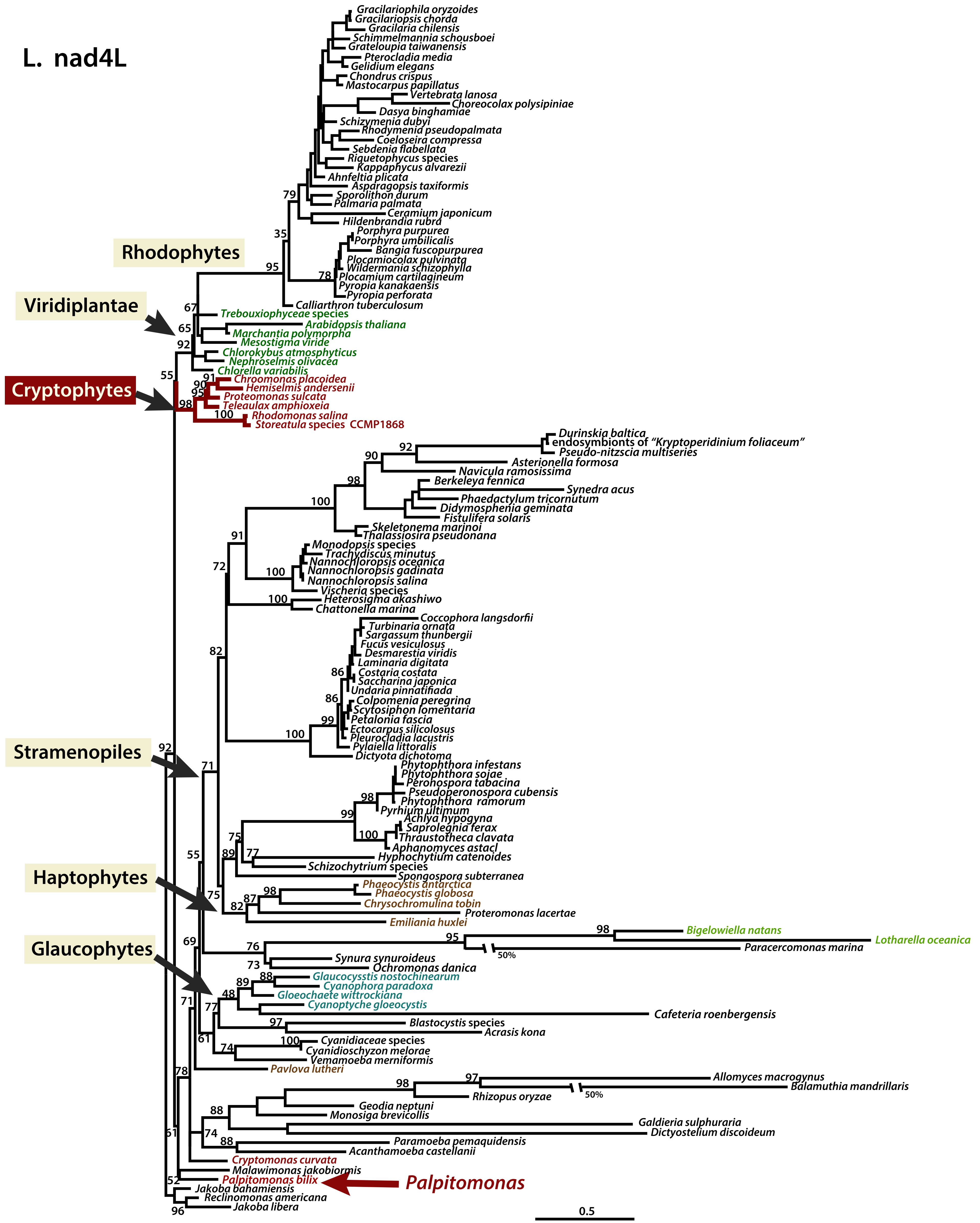
L. nad4L
Rhodophytes
Viridiplantae
Cryptophytes
Stramenopiles
Haptophytes
Glaucophytes
Gracilariophila oryzoides
Gracilariopsis chorda
Gracilaria chilensis
Schimmelmannia schousboei
Grateloupia taiwanensis
Pterocladia media
Gelidium elegans
Chondrus crispus
Mastocarpus papillatus
Vertebrata lanosa
Choreocolax polysipiniae
Dasya binghamiae
Schizymenia dubyi
Rhodymenia pseudopalmata
Coeloseira compressa
Sebdenia flabellata
Riquetophycus species
Kappaphycus alvarezii
Ahnfeltia plicata
Asparagopsis taxiformis
Sporolithon durum
Palmaria palmata
Ceramium japonicum
Hildenbrandia rubra
Porphyra purpurea
Porphyra umbilicalis
Bangia fuscopurpurea
Plocamiocolax pulvinata
Wildermania schizophylla
Plocamium cartilagineum
Pyropia kanakaensis
Pyropia perforata
Calliarthron tuberculosum
Trebouxiophyceae species
Arabidopsis thaliana
Marchantia polymorpha
Mesostigma viride
Chlorokybus atmosphyticus
Nephroselmis olivacea
Chlorella variabilis
Chroomonas placoidea
Hemiselmis andersenii
Proteomonas sulcata
Teleaulax amphioxeia
Rhodomonas salina
Storeatula species CCMP1868
Durinskia baltica
endosymbionts of "Kryptoperidinium foliaceum"
Pseudo-nitzscia multiseries
Asterionella formosa
Navicula ramosissima
Berkeleya fennica
Synedra acus
Phaedactylum tricornutum
Didymosphenia geminata
Fistulifera solaris
Skeletonema marinoi
Thalassiosira pseudonana
Monodopsis species
Trachydiscus minutus
Nannochloropsis oceanica
Nannochloropsis gadinata
Nannochloropsis salina
Vischeria species
Heterosigma akashiwo
Chattonella marina
Coccophora langsdorfii
Turbinaria ornata
Sargassum thunbergii
Fucus vesiculosus
Desmarestia viridis
Laminaria digitata
Costaria costata
Saccharina japonica
Undaria pinnatifiada
Colpomenia peregrina
Scytosiphon lomentaria
Petalonia fascia
Ectocarpus silicolosus
Pleurocladia lacustris
Pylaiella littoralis
Dictyota dichotoma
Phytophthora infestans
Phytophthora sojae
Peronospora tabacina
Pseudoperonospora cubensis
Phytophthora ramorum
Pyrhium ultimum
Achlya hypogyna
Saprolegnia ferax
Thraustotheca clavata
Aphanomyces astaci
Hyphochytium catenoides
Schizochytrium species
Spongospora subterranea
Phaeocystis antarctica
Phaeocystis globosa
Chrysochromulina tobin
Proteromonas lacertae
Emiliania huxlei
Bigelowiella natans
Lotharella oceanica
Paracercomonas marina
50%
Synura synuroideus
Ochromonas danica
Glaucocysstis nostochinearum
Cyanophora paradoxa
Gloeochaete wittrockiana
Cyanoptyche gloeocystis
Cafeteria roenbergensis
Blastocystis species
Acrasis kona
Cyanidiaceae species
Cyanidioschyzon melorae
Vemamoeba merniformis
Pavlova lutheri
Allomyces macrogynus
Balamuthia mandrillaris
Rhizopus oryzae
50%
Geodia neptuni
Monosiga brevicollis
Galdieria sulphuraria
Dictyostelium discoideum
Paramoeba pemaquidensis
Acanthamoeba castellanii
Cryptomonas curvata
Malawimonas jakobiormis
Palpitomonas bilix
Palpitomonas
Jakoba bahamiensis
Reclinomonas americana
Jakoba libera
0.5

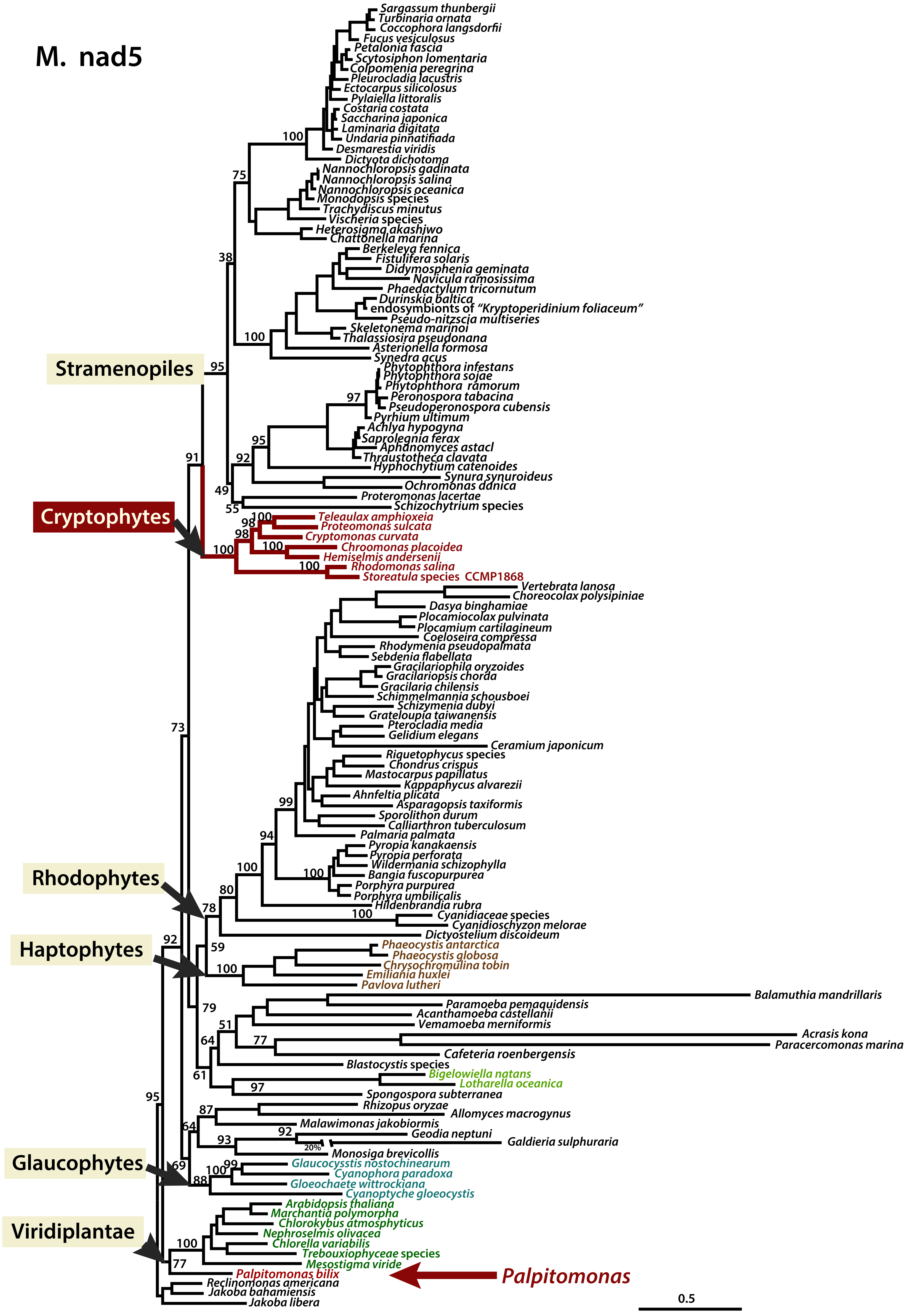

M. nad5
Sargassum thunbergii
Turbinaria ornata
Coccophora langsdorfii
Fucus vesiculosus
Petalonia fascia
Scytosiphon lomentaria
Colpomenia peregrina
Pleurocladia lacustris
Ectocarpus silicolosus
Pylaiella littoralis
Costaria costata
Saccharina japonica
Laminaria digitata
Undaria pinnatifiada
Desmarestia viridis
Dictyota dichotoma
Nannochloropsis gadinata
Nannochloropsis salina
Nannochloropsis oceanica
Monodopsis species
Trachydiscus minutus
Vischeria species
Heterosigma akashiwo
Chattonella marina
Berkeleya fennica
Fistulifera solaris
Didymosphenia geminata
Navicula ramosissima
Phaedactylum tricornutum
Durinskia baltica
endosymbionts of "Kryptoperidinium foliaceum"
Pseudo-nitzscia multiseries
Skeletonema marinoi
Thalassiosira pseudonana
Asterionella formosa
Synedra acus
Phytophthora infestans
Phytophthora sojae
Phytophthora ramorum
Peronospora tabacina
Pseudoperonospora cubensis
Pyrhium ultimum
Achlya hypogyna
Saprolegnia ferax
Aphanomyces astacl
Thraustotheca clavata
Hyphochytium catenoides
Synura synuroideus
Ochromonas danica
Proteromonas lacertae
Schizochytrium species
Stramenopiles
Cryptophytes
Teleaulax amphioxeia
Proteomonas sulcata
Cryptomonas curvata
Chroomonas placoidea
Hemiselmis andersenii
Rhodomonas salina
Storeatula species CCMP1868
Vertebrata lanosa
Choreocolax polysipiniae
Dasya binghamiae
Plocamiocolax pulvinata
Plocamium cartilagineum
Coeloseira compressa
Rhodymenia pseudopalmata
Sebdenia flabellata
Gracilariophila oryzoides
Gracilariopsis chorda
Gracilaria chilensis
Schimmelmannia schousboei
Schizymenia dubyi
Grateloupia taiwanensis
Pterocladia media
Gelidium elegans
Ceramium japonicum
Riquetophycus species
Chondrus crispus
Mastocarpus papillatus
Kappaphycus alvarezii
Ahnfeltia plicata
Asparagopsis taxiformis
Sporolithon durum
Calliarthron tuberculosum
Palmaria palmata
Pyropia kanakaensis
Pyropia perforata
Wildermania schizophylla
Bangia fuscopurpurea
Porphyra purpurea
Porphyra umbilicalis
Hildenbrandia rubra
Cyanidiaceae species
Cyanidioschyzon melorae
Dictyostelium discoideum
Rhodophytes
Haptophytes
Phaeocystis antarctica
Phaeocystis globosa
Chrysochromulina tobin
Emiliania huxlei
Pavlova lutheri
Balamuthia mandrillaris
Paramoeba pemaquidensis
Acanthamoeba castellanii
Vemamoeba merniformis
Acrasis kona
Paracercomonas marina
Cafeteria roenbergensis
Blastocystis species
Bigelowiella natans
Lotharella oceanica
Spongospora subterranea
Rhizopus oryzae
Allomyces macrogynus
Malawimonas jakobiormis
Geodia neptuni
Galdieria sulphuraria
20%
Monosiga brevicollis
Glaucophytes
Glaucocysstis nostochinearum
Cyanophora paradoxa
Gloeochaete wittrockiana
Cyanoptyche gloeocystis
Viridiplantae
Arabidopsis thaliana
Marchantia polymorpha
Chlorokybus atmosphyticus
Nephroselmis olivacea
Chlorella variabilis
Trebouxiophyceae species
Mesostigma viride
Palpitomonas bilix
Palpitomonas
Reclinomonas americana
Jakoba bahamiensis
Jakoba libera
0.5

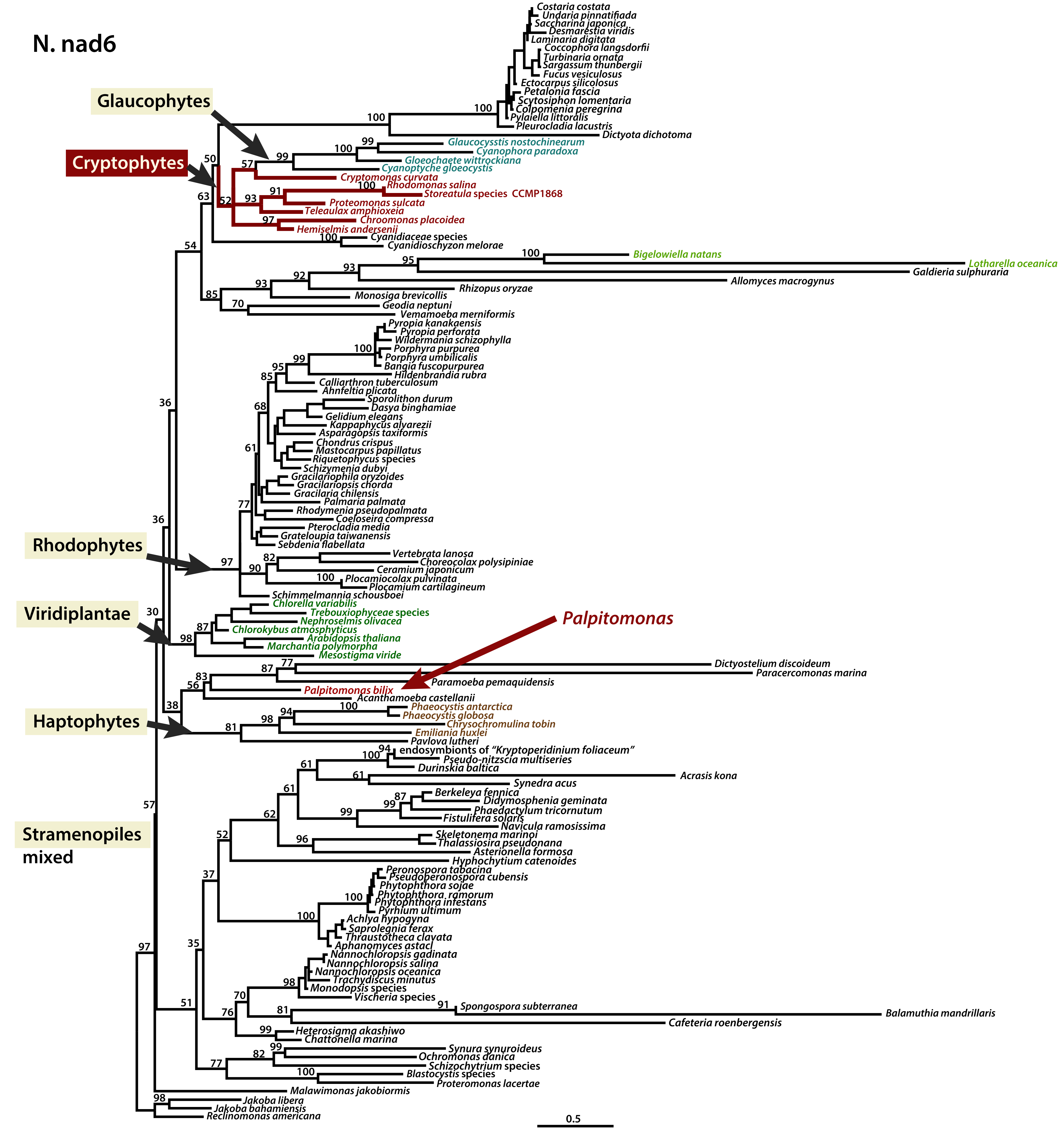

N. nad6
Glaucophytes
Cryptophytes
Rhodophytes
Viridiplantae
Haptophytes
Stramenopiles mixed
Palpitomonas
Costaria costata
Undaria pinnatifiada
Saccharina japonica
Desmarestia viridis
Laminaria digitata
Coccophora langsdorfii
Turbinaria ornata
Sargassum thunbergii
Fucus vesiculosus
Ectocarpus silicolosus
Petalonia fascia
Scytosiphon lomentaria
Colpomenia peregrina
Pylaiella littoralis
Pleurocladia lacustris
Dictyota dichotoma
Glaucocysstis nostochinearum
Cyanophora paradoxa
Gloeochaete wittrockiana
Cyanoptyche gloeocystis
Cryptomonas curvata
Rhodomonas salina
Storeatula species CCMP1868
Proteomonas sulcata
Teleaulax amphioxeia
Chroomonas placoidea
Hemiselmis andersenii
Cyanidiaceae species
Cyanidioschyzon melorae
Bigelowiella natans
Lotharella oceanica
Galdieria sulphuraria
Allomyces macrogynus
Rhizopus oryzae
Monosiga brevicollis
Geodia neptuni
Vemamoeba merniformis
Pyropia kanakaensis
Pyropia perforata
Wildermania schizophylla
Porphyra purpurea
Porphyra umbilicalis
Bangia fuscopurpurea
Hildenbrandia rubra
Calliarthron tuberculosum
Ahnfeltia plicata
Sporolithon durum
Dasya binghamiae
Gelidium elegans
Kappaphycus alvarezii
Asparagopsis taxiformis
Chondrus crispus
Mastocarpus papillatus
Riquetophycus species
Schizymenia dubyi
Gracilariophila oryzoides
Gracilariopsis chorda
Gracilaria chilensis
Palmaria palmata
Rhodymenia pseudopalmata
Coeloseira compressa
Pterocladia media
Grateloupia taiwanensis
Sebdenia flabellata
Vertebrata lanosa
Choreocolax polysipiniae
Ceramium japonicum
Plocamiocolax pulvinata
Plocamium cartilagineum
Schimmelmannia schousboei
Chlorella variabilis
Trebouxiophyceae species
Nephroselmis olivacea
Chlorokybus atmosphyticus
Arabidopsis thaliana
Marchantia polymorpha
Mesostigma viride
Dictyostelium discoideum
Paracercomonas marina
Paramoeba pemaquidensis
Palpitomonas bilix
Acanthamoeba castellanii
Phaeocystis antarctica
Phaeocystis globosa
Chrysochromulina tobin
Emiliania huxlei
Pavlova lutheri
endosymbionts of "Kryptoperidinium foliaceum"
Pseudo-nitzscia multiseries
Durinskia baltica
Acrasis kona
Synedra acus
Berkeleya fennica
Didymosphenia geminata
Phaedactylum tricornutum
Fistulifera solaris
Navicula ramosissima
Skeletonema marinoi
Thalassiosira pseudonana
Asterionella formosa
Hyphochytium catenoides
Peronospora tabacina
Pseudoperonospora cubensis
Phytophthora sojae
Phytophthora ramorum
Phytophthora infestans
Pyrhium ultimum
Achlya hypogyna
Saprolegnia ferax
Thraustotheca clavata
Aphanomyces astaci
Nannochloropsis gadinata
Nannochloropsis salina
Nannochloropsis oceanica
Trachydiscus minutus
Monodopsis species
Vischeria species
Spongospora subterranea
Balamuthia mandrillaris
Cafeteria roenbergensis
Heterosigma akashiwo
Chattonella marina
Synura synuroideus
Ochromonas danica
Schizochytrium species
Blastocystis species
Proteromonas lacertae
Malawimonas jakobiormis
Jakoba libera
Jakoba bahamiensis
Reclinomonas americana
0.5

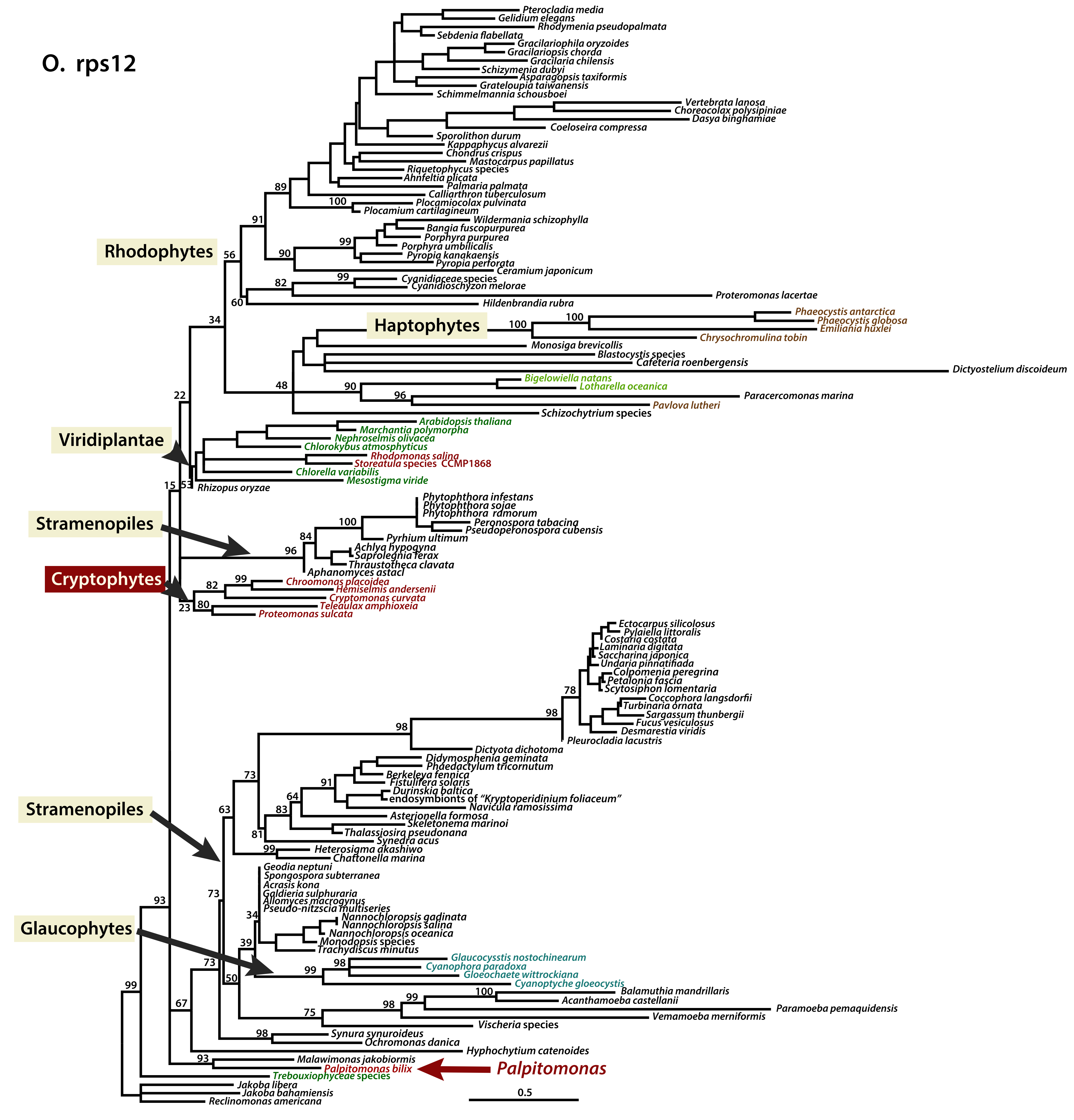
O. rps12
Rhodophytes
Haptophytes
Viridiplantae
Stramenopiles
Cryptophytes
Stramenopiles
Glaucophytes
Palpitomonas
Pterocladia media
Gelidium elegans
Rhodymenia pseudopalmata
Sebdenia flabellata
Gracilariophila oryzoides
Gracilariopsis chorda
Gracilaria chilensis
Schizymenia dubyi
Asparagopsis taxiformis
Grateloupia taiwanensis
Schimmelmannia schousboei
Vertebrata lanosa
Choreocolax polysipiniae
Dasya binghamiae
Coeloseira compressa
Sporolithon durum
Kappaphycus alvarezii
Chondrus crispus
Mastocarpus papillatus
Riquetophycus species
Ahnfeltia plicata
Palmaria palmata
Calliarthron tuberculosum
Plocamiocolax pulvinata
Plocamium cartilagineum
Wildermania schizophylla
Bangia fuscopurpurea
Porphyra purpurea
Porphyra umbilicalis
Pyropia kanakaensis
Pyropia perforata
Ceramium japonicum
Cyanidiaceae species
Cyanidioschyzon melorae
Proteromonas lacertae
Hildenbrandia rubra
Phaeocystis antarctica
Phaeocystis globosa
Emiliania huxlei
Chrysochromulina tobin
Monosiga brevicollis
Blastocystis species
Cafeteria roenbergensis
Dictyostelium discoideum
Bigelowiella natans
Lotharella oceanica
Paracercomonas marina
Pavlova lutheri
Schizochytrium species
Arabidopsis thaliana
Marchantia polymorpha
Nephroselmis olivacea
Chlorokybus atmosphyticus
Rhodomonas salina
Storeatula species CCMP1868
Chlorella variabilis
Mesostigma viride
Rhizopus oryzae
Phytophthora infestans
Phytophthora sojae
Phytophthora ramorum
Peronospora tabacina
Pseudoperonospora cubensis
Pyrhium ultimum
Achlya hypogyna
Saprolegnia ferax
Thraustotheca clavata
Aphanomyces astaci
Chroomonas placoidea
Hemiselmis andersenii
Cryptomonas curvata
Teleaulax amphioxeia
Proteomonas sulcata
Ectocarpus silicolosus
Pylaiella littoralis
Costaria costata
Laminaria digitata
Saccharina japonica
Undaria pinnatifiada
Colpomenia peregrina
Petalonia fascia
Scytosiphon lomentaria
Coccophora langsdorfii
Turbinaria ornata
Sargassum thunbergii
Fucus vesiculosus
Desmarestia viridis
Pleurocladia lacustris
Dictyota dichotoma
Didymosphenia geminata
Phaedactylum tricornutum
Berkeleya fennica
Fistulifera solaris
Durinskia baltica
endosymbionts of "Kryptoperidinium foliaceum"
Navicula ramosissima
Asterionella formosa
Skeletonema marinoi
Thalassiosira pseudonana
Synedra acus
Heterosigma akashiwo
Chattonella marina
Geodia neptuni
Spongospora subterranea
Acrasis kona
Galdieria sulphuraria
Allomyces macrogynus
Pseudo-nitzscia multiseries
Nannochloropsis gadinata
Nannochloropsis salina
Nannochloropsis oceanica
Monodopsis species
Trachydiscus minutus
Glaucocysstis nostochinearum
Cyanophora paradoxa
Gloeochaete wittrockiana
Cyanoptyche gloeocystis
Balamuthia mandrillaris
Acanthamoeba castellanii
Paramoeba pemaquidensis
Vemamoeba merniformis
Vischeria species
Synura synuroideus
Ochromonas danica
Hyphochytium catenoides
Malawimonas jakobiormis
Palpitomonas bilix
Trebouxiophyceae species
Jakoba libera
Jakoba bahamiensis
Reclinomonas americana
0.5

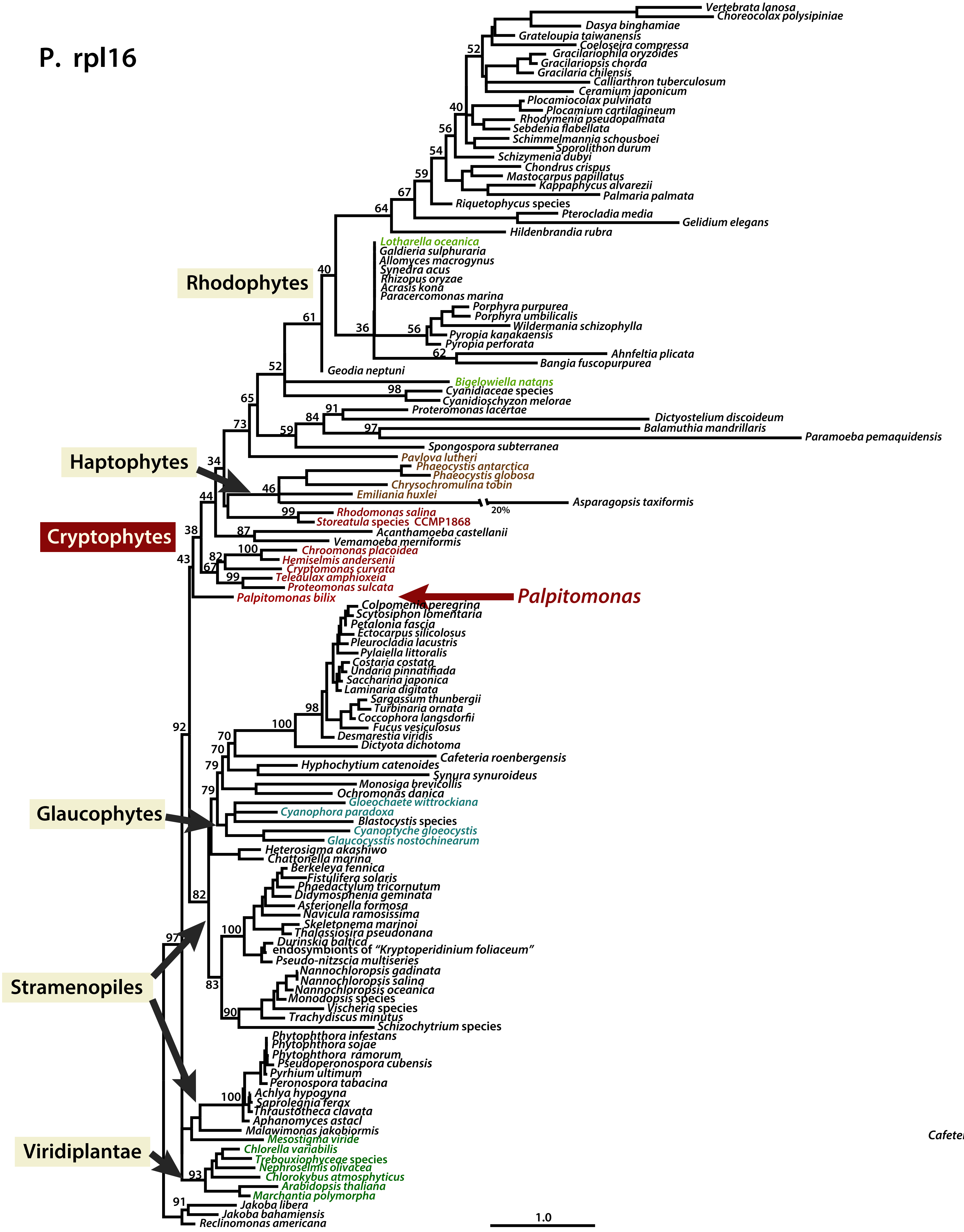

P. rpl16
Rhodophytes
Haptophytes
Cryptophytes
Palpitomonas
Glaucophytes
Stramenopiles
Viridiplantae
Vertebrata lanosa
Choreocolax polysipiniae
Dasya binghamiae
Grateloupia taiwanensis
Coeloseira compressa
Gracilariophila oryzoides
Gracilariopsis chorda
Gracilaria chilensis
Calliarthron tuberculosum
Ceramium japonicum
Plocamiocolax pulvinata
Plocamium cartilagineum
Rhodymenia pseudopalmata
Sebdenia flabellata
Schimmelmannia schousboei
Sporolithon durum
Schizymenia dubyi
Chondrus crispus
Mastocarpus papillatus
Kappaphycus alvarezii
Palmaria palmata
Riquetophycus species
Pterocladia media
Gelidium elegans
Hildenbrandia rubra
Lotharella oceanica
Galdieria sulphuraria
Allomyces macrogynus
Synedra acus
Rhizopus oryzae
Acrasis kona
Paracercomonas marina
Porphyra purpurea
Porphyra umbilicalis
Wildermania schizophylla
Pyropia kanakaensis
Pyropia perforata
Ahnfeltia plicata
Bangia fuscopurpurea
Geodia neptuni
Bigelowiella natans
Cyanidiaceae species
Cyanidioschyzon melorae
Proteromonas lacertae
Dictyostelium discoideum
Balamuthia mandrillaris
Paramoeba pemaquidensis
Spongospora subterranea
Pavlova lutheri
Phaeocystis antarctica
Phaeocystis globosa
Chrysochromulina tobin
Emiliania huxlei
Asparagopsis taxiformis
20%
Rhodomonas salina
Storeatula species CCMP1868
Acanthamoeba castellanii
Vemamoeba merniformis
Chroomonas placoidea
Hemiselmis andersenii
Cryptomonas curvata
Teleaulax amphioxeia
Proteomonas sulcata
Palpitomonas bilix
Colpomenia peregrina
Scytosiphon lomentaria
Petalonia fascia
Ectocarpus silicolosus
Pleurocladia lacustris
Pylaiella littoralis
Costaria costata
Undaria pinnatifiada
Saccharina japonica
Laminaria digitata
Sargassum thunbergii
Turbinaria ornata
Coccophora langsdorfii
Fucus vesiculosus
Desmarestia viridis
Dictyota dichotoma
Cafeteria roenbergensis
Hyphochytium catenoides
Synura synuroideus
Monosiga brevicollis
Ochromonas danica
Gloeochaete wittrockiana
Cyanophora paradoxa
Blastocystis species
Cyanoptyche gloeocystis
Glaucocysstis nostochinearum
Heterosigma akashiwo
Chattonella marina
Berkeleya fennica
Fistulifera solaris
Phaedactylum tricornutum
Didymosphenia geminata
Asterionella formosa
Navicula ramosissima
Skeletonema marinoi
Thalassiosira pseudonana
Durinskia baltica
endosymbionts of "Kryptoperidinium foliaceum"
Pseudo-nitzscia multiseries
Nannochloropsis gadinata
Nannochloropsis salina
Nannochloropsis oceanica
Monodopsis species
Vischeria species
Trachydiscus minutus
Schizochytrium species
Phytophthora infestans
Phytophthora sojae
Phytophthora ramorum
Pseudoperonospora cubensis
Pyrhium ultimum
Peronospora tabacina
Achlya hypogyna
Saprolegnia ferax
Thraustotheca clavata
Aphanomyces astaci
Malawimonas jakobiormis
Mesostigma viride
Chlorella variabilis
Trebouxiophyceae species
Nephroselmis olivacea
Chlorokybus atmosphyticus
Arabidopsis thaliana
Marchantia polymorpha
Jakoba libera
Jakoba bahamiensis
Reclinomonas americana
1.0
Cafeter
